# Screening bacterial effectors and human virus proteins in yeast to identify host factors driving tombusvirus RNA recombination: a role for autophagy and membrane phospholipid content

**DOI:** 10.1128/jvi.01661-24

**Published:** 2025-05-27

**Authors:** Judit Pogany, Jun-ichi Inaba, Yuyan Liu, Peter D. Nagy

**Affiliations:** 1 Department of Plant Pathology, University of Kentucky 4530, Lexington, Kentucky, USA; Iowa State University, Ames, Iowa, USA

**Keywords:** RNA recombination, virus replication, tomato bushy stunt virus, host-virus interaction, SARS-CoV-2, HMPV, *Legionella pneumophila*, yeast, autophagy, phospholipids

## Abstract

Recombination in RNA viruses contributes to virus evolution and rapid emergence of new viral variants that helps evade host’s antiviral strategies. Host factors play important but poorly characterized roles in viral RNA recombination. The authors expressed *Legionella* bacterium effector proteins and SARS-CoV-2 and human metapneumovirus (HMPV) proteins in yeast to test their effects on tomato bushy stunt virus (TBSV) RNA recombination. The identified 16 *Legionella* effectors, six SARS-CoV-2, and two HMPV proteins affecting TBSV recombination likely target shared host factors with TBSV. Among the targets of the effectors/viral proteins was the autophagy pathway. Inhibition of autophagy by expression of RavZ and LegA9 *Legionella* effectors reduced the production of TBSV recombinants in yeast and plants. Induction of autophagy by rapamycin, via nitrogen starvation of yeast or overexpression of ATG2 lipid transfer protein, led to enhanced viral RNA recombination. Using *in vitro* TBSV replicase assembly on giant unilamellar vesicles confirmed the critical role of phosphatidylethanolamine in RNA recombination. We suggest that the pro-recombination role of co-opted autophagy is to provide abundant phospholipids for viral replication organelle biogenesis. Overall, this work highlights the critical roles of membrane phospholipids and lipid context in the regulation of viral RNA recombination. We show that SARS-CoV-2 N and HMPV M2-1 proteins enhance TBSV RNA replication and recombination by protecting the viral RNAs from host Xrn1 5´−3´ exoribonuclease in yeast. Altogether, the novel strategy of using TBSV as a cellular system sensor might assist in the identification of novel functional targets of various viral and bacterial effectors in yeast.

**IMPORTANCE:**

Positive-strand (+)RNA viruses replicate in the cytosol of infected cells by exploiting cellular proteins and resources that frequently lead to diseases. Virus replication results in the generation of viral RNA recombinants that contribute to the emergence of new viral variants and adaptation to new hosts. The authors expressed *Legionella* bacterium effector proteins, SARS-CoV-2 and human metapneumovirus proteins in yeast to test their effects on tomato bushy stunt virus (TBSV) RNA recombination. This novel approach revealed that *Legionella* effectors and heterologous viral proteins target shared host factors with TBSV, including the autophagy pathway. *In vitro* approach revealed that the pro-recombination role of co-opted autophagy is to provide abundant phospholipids for viral replication. SARS-CoV-2 nucleocapsid protein and human metapneumovirus M2-1 protein are shown to enhance TBSV RNA replication and recombination by protecting the viral RNAs from host Xrn1 5´−3´ exoribonuclease in yeast. Thus, the TBSV/yeast system can be used as a cellular system sensor to find new functions of heterologous viral proteins.

## INTRODUCTION

Positive-strand (+)RNA viruses replicate in the cytosol of infected cells by exploiting cellular proteins and resources that frequently lead to diseases. These viruses extensively reprogram host metabolism, hijack cellular organelles, and rewire cellular pathways to build viral replication organelles (VROs) that lead to the production of numerous virus progeny (1–10). (+)RNA virus replication also results in the generation of viral RNA recombinants that are a major driver of (+)RNA virus evolution, emergence of new viral variants, and adaptation to new hosts (11–13). Viral RNA recombination occurs when two or more noncontiguous segments of the same RNA or two separate RNAs are joined together (14–17). Viral recombination has been shown to result in mutations, sequence insertions, duplications, deletions, and sequence rearrangements (16, 18–22). Many RNA recombination events are likely deleterious to viral infectivity, but they could also generate new functional cis-acting RNA elements or novel coding sequences, thus increasing the sequence space probed by viruses (23). RNA recombination could also occur between different, sometimes unrelated viruses (interviral recombination) to help “jump” to new hosts (24, 25). Interestingly, RNA recombination also contributes to the repair of truncated or mutated viral RNA genomes. This feature leads to increased infectivity of (+)RNA viruses, which are prone to high-frequency mutations during RNA replication (13, 26).

Viral RNA recombination and its importance have been well-documented for many viruses, including SARS-CoV-2 (12, 27, 28). Most viral RNA recombination events are driven by the template switching mechanism driven by the viral RNA-dependent RNA polymerase (RdRp) (14, 29–36). However, the hosts also play a significant role in modulating RNA recombination events. This was amply demonstrated with tomato bushy stunt virus (TBSV), a tombusvirus, based on the development of various unique approaches including the use of yeast (*Saccharomyces cerevisiae*) model host (37–41). Systematic genome-wide screens with gene-deletion libraries or temperature-sensitive mutants in yeast have identified over 80 host genes that affected TBSV RNA recombination (42–48).

Four different cellular pathways have been characterized for their roles in TBSV recombination. The first pathway (i) includes cellular endo- and exoribonucleases that cleave the viral RNAs, which are then used by the viral replicase as templates for template-switching recombination (45, 49, 50). The best characterized member of this pathway is the cytosolic Xrn1 5´−3´ exoribonuclease (Xrn4 in plants) (49, 51–53). (ii) The amount of cytosolic Mn^++^ is also critical during viral RNA recombination. This was demonstrated by deletion of yeast Pmr1 (ATP2C1 in humans) Ca^++^/Mn^++^ pump, which led to enhanced template-switching activity of the viral replicase (24, 46). (iii) Cellular co-opted DEAD-box helicases also affect viral RNA synthesis by locally unwinding viral RNA structures and controlling viral RdRp:RNA template interactions (54–57). (iv) The co-opted actin network in collaboration with Rpn11 proteasomal deubiquitinase plays an integral part in viral replicase complex (VRC) assembly, and these factors suppress TBSV RNA recombination by facilitating proper VRC assembly/function (47, 58–60). The above proteins and pathways, however, only represent a fraction of host factors identified in the yeast screens, suggesting that our knowledge on the role of the host in viral RNA recombination is incomplete.

TBSV codes for p33 and p92^pol^ replication proteins that are directly translated from the genomic (g)RNA. The p92^pol^ is a translational readthrough product of p33 open reading frame (ORF) (61–63). The RdRp function of p92^pol^ depends on viral and host components, such as heat shock protein 70, translation elongation factors, and DEAD-box helicases (37, 64–69). The abundant p33 is the master regulator of TBSV replication. P33 has an RNA chaperone function, and it has a key role in the recruitment of viral (+)RNA template for replication and in the assembly of the membrane-bound VRCs (66, 70–75).

In the current work, to deepen our understanding of the host in viral RNA recombination, we have performed a novel systematic screen with TBSV based on a library of *Legionella* effectors that affect cellular functions. This screen has led to the identification of 16 *Legionella* effectors affecting TBSV RNA recombination. In addition, we screened viral proteins expressed by the unrelated SARS-CoV-2 and human metapneumovirus (HMPV) to identify common host targets with TBSV in yeast. This led to the identification of six SARS-CoV-2 and two HMPV proteins that targeted host factors also affecting TBSV replication or RNA recombination. Interestingly, the cellular autophagy pathway was among the most frequent “hits” in the above screens.

Autophagy functions in maintaining cellular homeostasis and defense against invading pathogens in plants and animals. Autophagy is performed by autophagy-related proteins (ATGs) that assemble the double-membrane autophagosome and recruit cargo proteins or damaged organelles, followed by degradation in vacuoles/lysosomes (76–80). Autophagy has an antiviral role via degradation of viral proteins or virions. Several viruses, however, block autophagy or exploit autophagy to degrade host defense factors and support virus replication (81–89). Autophagy also targets plant viruses, and it is important in antiviral innate immunity (90–96). Several plant viruses, including TBSV, also exploit autophagy for pro-viral functions (86, 97–99). TBSV sequesters ATG8f autophagy core protein and NBR1 cargo receptor protein into condensate substructures within the membranous VROs to inhibit autophagy flux, and also hijacks the phospholipid-rich autophagy membrane for VRO biogenesis (99, 100). However, the role of autophagy in viral RNA recombination is poorly understood.

Therefore, we have chosen the autophagy pathway to provide evidence on its role in TBSV recombination in yeast and plants. We show that inhibition of the autophagy process reduced TBSV RNA recombinantion, whereas induction of autophagy increased the occurrence of TBSV RNA recombinants. Overexpression of ATG2 autophagy-related bulk lipid transfer protein enhanced TBSV recombination, pointing toward the possible role of phospholipids in TBSV RNA recombination. Indeed, using our *in vitro* approach with giant unilamellar vesicles (GUVs), we confirmed the critical roles of selected phospholipids, such as phosphatidylethanolamine (PE), in RNA recombination. The phospholipids are mostly provided by the co-opted autophagy and the ATG2 autophagy-related bulk lipid transfer protein (99, 101, 102). Interestingly, more detailed analysis revealed that the SARS-CoV-2 N nucleocapsid protein and HMPV M2-1 protein may protect TBSV RNAs from the host Xrn1 5´−3´ exoribonuclease, which is a major suppressor of TBSV replication and RNA recombination in yeast. Overall, the bacterial effectors and heterologous viral proteins helped define the novel function of cellular autophagy in viral RNA recombination. Moreover, we show that the novel strategy of using TBSV as a cellular system sensor might assist in the identification of novel functional targets of various viral and bacterial effectors in yeast.

## RESULTS

### Rationale

**Fig 1:**
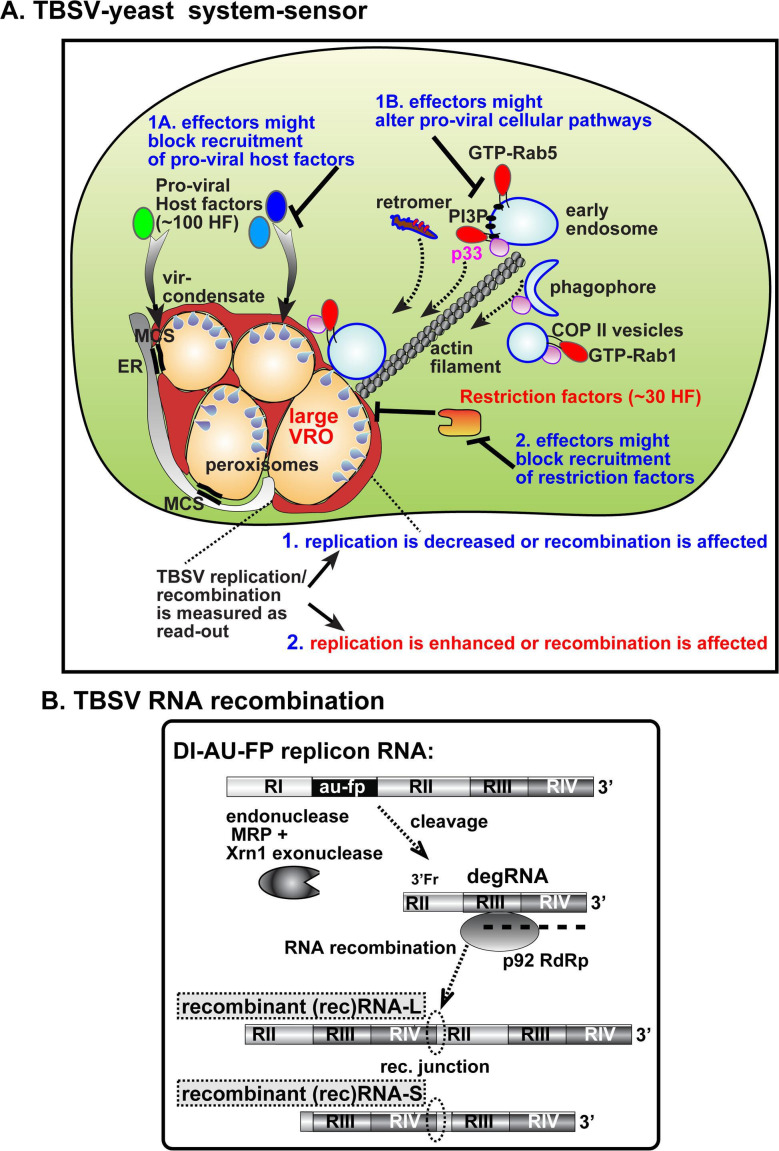
A scheme on the use of TBSV-yeast as a cellular system sensor. (**A**) Expression of bacterial effectors or heterologous viral proteins might target the same host factors or cellular pathways that are exploited by TBSV. TBSV is known to hijack ~100 host factors (target #1A) and subvert Rab1-decorated COPII vesicles, Rab5-decorated early endosomes, Rab7-associated retromer complex, and phagophores (autophagy pathway) (target #1B) with the assistance from the co-opted actin filaments to build large VROs. The VROs are formed from hijacked clustered peroxisomes and ER membranes, which form membrane contact sites (vMCS, black double lines) for sterol transfer to VROs. The membranous VROs also contain condensate substructures (reddish). Moreover, host restriction factors (~30 proteins) inhibit TBSV replication (target #2). The effect of heterologous protein/effector expression in yeast might target the co-opted proteins and pathways, thus causing changes in TBSV replication and recombination, which are measured as a read-out of the assay. (**B**) The highly recombinogenic TBSV DI-AU-FP replicon (rep)RNA replicates in yeast in the presence of p33 and p92^pol^ replication proteins, but it could also form RNA recombinants via template switching by the RdRp, which are schematically shown. Partially degraded TBSV (deg)RNAs could serve as donor or acceptor templates during recombination events. The recombinant (rec)RNAs are different in sizes based on the recombination junction sites as shown. Note that the replicon RNA does not carry a selection marker.

Effectors of bacterial pathogens and viral proteins (pathogenicity proteins), which are targeted to/produced inside the host cells, are known to modify various cellular proteins and lipids or rewire cellular pathways to facilitate pathogen propagation/replication (103–106). Our hypothesis is that selected effector proteins, when expressed in eukaryotic cells, might target/modify the same cellular proteins or cellular pathways that are also exploited/co-opted by TBSV. Therefore, we hypothesize that various pathogen effectors could compete with TBSV for common (shared) resources, thus affecting TBSV replication as we have shown earlier (107) and possibly TBSV RNA recombination in yeast (Fig. 1). Alternatively, the activity of yeast antiviral proteins, such as nucleases and cyclophilins (48, 50, 108), etc., could be inhibited by effectors/viral proteins, which might lead to altered TBSV replication or recombination. In other words, alteration in TBSV replication and recombination caused by the expression of the heterologous effectors in yeast could be useful to identify cellular targets and functions of pathogen effectors, in combination with revealing the biology of TBSV replication and recombination. To test this working model, we screened *Legionella* bacterium effectors and SARS-CoV-2 and HMPV proteins for their effects on TBSV RNA recombination based on the yeast model host.

### Screening of a library of *Legionella* bacterium effectors to identify host pathways and host genes that alter tombusvirus RNA recombination in yeast

To identify host factors/pathways affecting tombusvirus RNA recombination, here, we have performed a novel screen with a library of *Legionella* effectors in yeast. The original library contains 302 effectors (provided by Drs. B. Lindenbach and C. Roy), which are delivered to human cells to facilitate the infection cycle of bacteria (106). However, we selected only 113 effectors, which were previously found to affect TBSV RNA replication (107) and/or having known functions in cells (109, 110). These *Legionella* effectors alter evolutionary conserved cellular processes/pathways, making them suitable to probe virus-host interaction.

We have used the wild type (WT) yeast strain (BY4741) to test the effect of heterologous proteins on TBSV RNA recombination. The RNA recombination system is based on the highly recombinogenic DI-AU-FP replicon (rep)RNA (Fig. 1B) (111), which is expressed together with the tombusvirus p33 and p92^pol^ replication proteins to initiate TBSV repRNA replication and recombination in yeast (45, 112). The TBSV repRNA-derived recombinants are generated via a template-switching mechanism by the viral replicase (31, 46, 50, 113). RNA recombination events are facilitated by cleavages of the viral repRNA templates by cellular endo- and exoribonucleases that lead to the generation of different-sized RNA recombinants (schematically shown in Fig. 1B) (38, 44–46, 49, 50, 52).

**Fig 2:**
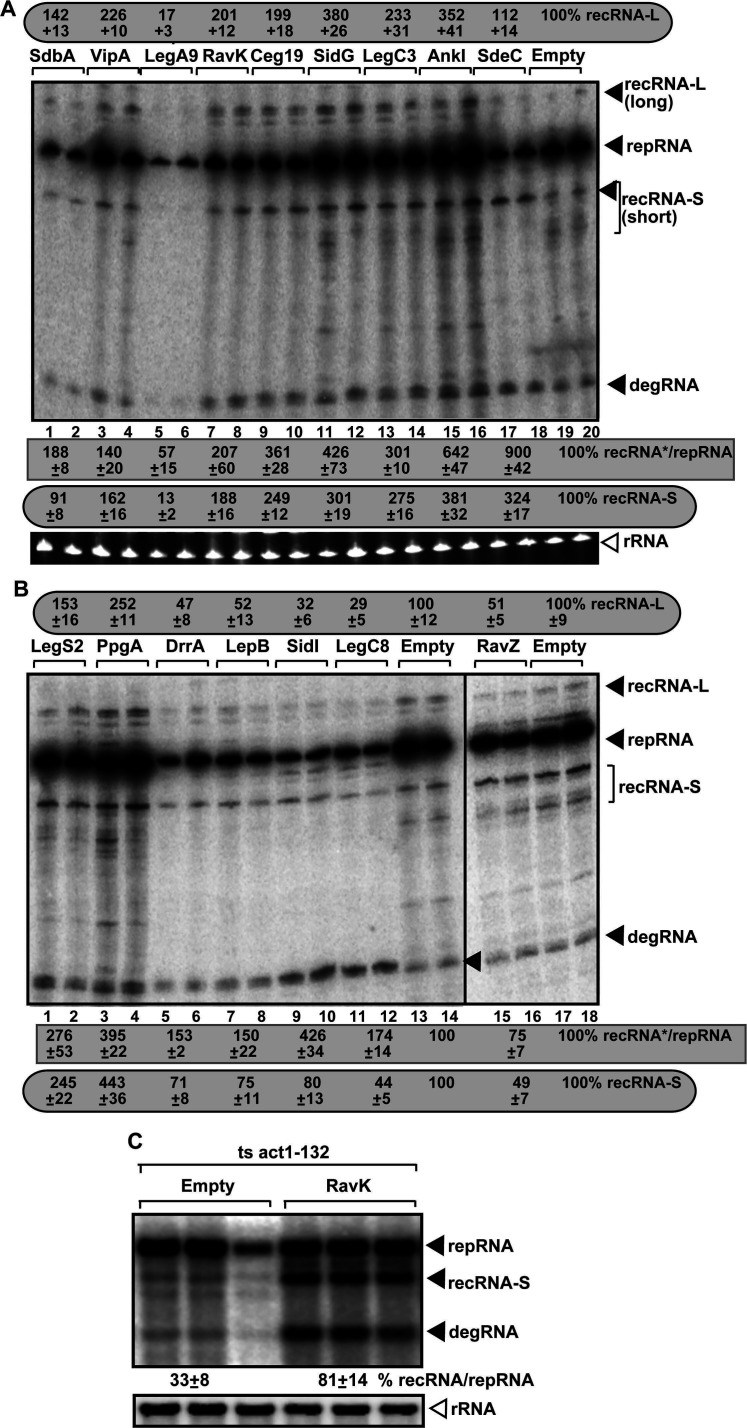
Expression of *Legionella* effectors alters TBSV RNA recombination in yeast. (**A, B**) The effect of *Legionella* effectors on TBSV recRNA production was measured by northern blot 36 h after initiation of TBSV replication via expression of p33, p92^pol^ replication proteins, and DI-AU-FP repRNA in BY4741 yeast. The accumulation level of recRNA-L and recRNA-S was taken as 100% in yeast not expressing effectors. Note that we also calculated the ratio of recRNA (either recRNA-S or recRNA-L, depending on the largest change) per replicon repRNA. Each sample is obtained from independent yeast colonies. Bottom panel: ethidium-bromide-stained gel with ribosomal RNA, as a loading control. Each experiment was repeated. (**C**) The effect of expression of *Legionella* RavK effector on accumulation level of recRNA in comparison to the replicon RNA in act1-132^ts^ yeast cultured at a semi-persistent temperature (29°C for 42 h). Northern blot analysis was used to detect replicon repRNA, recRNA, and the partially degraded repRNA (called degRNA) and the ribosomal RNA (rRNA). Each experiment was repeated.

After separately expressing 113 *Legionella* effectors from expression plasmids in WT yeast, which replicated TBSV replicon (rep)RNA, we performed northern blot analyses to measure TBSV recombinant (rec)RNA accumulation and repRNA replication level in yeast (107). The primary screen in high-throughput format has led to the identification of 40 effectors affecting TBSV RNA recombination by ~25% or more. To confirm the primary screen results, we tested the effects of *Legionella* effectors in yeast by measuring TBSV recombinant (rec)RNA accumulation (Fig. 2). In sum, 16 of the retested effectors affected TBSV recombination by ~40% or more, validating the robustness of our approach (Table 1). Interestingly, seven of the effectors also affected TBSV replication in yeast (Table 1) (107), suggesting that RNA recombination might be affected by host factors, which overlap with TBSV replication, but other host components might uniquely target RNA recombination.

**TABLE 1:** Identified *Legionella* effector proteins affecting TBSV RNA recombination in yeast^*a*^

ORF	Effector name	Function	Reference
lpg0275	SdbA^*b*^	Structural similarity with glycosyltransferases	(114)
lpg0390	VipA^*b*^	Actin nucleator, it localizes to the early endosomes	(115)
lpg0402	LegA9	Regulation of autophagy	(116)
lpg0969	RavK^*b*^	Disruptor of host cytoskeletal structure by cleaving Actin	(117)
pg1121	Ceg19	Subversion of vesicle trafficking	(109)
lpg1355	SidG	Unknown function	(118)
lpg1683	RavZ^*b*^	Binding to PI3P and high-curvature membranes, leading to autophagy inhibition, targets ATG8	(119, 120)
lpg1701	LegC3	Coiled-coil motifs (Q-SNARE-like), targets VAM4, subversion of vesicle trafficking, modulate membrane fusion	(121)
lpg1718	AnkI	Ankyrin repeat, modification of host chromatin, altered gene expression, targets H3K4	(122)
lpg2153	SdeC	Catalyze phosphoribosyl-linked ubiquitination (pR-Ub) of host targets	(123)
lpg2176	LegS2	Sphingosine-1-phosphate lyase, modulates autophagy	(124)
lpg2224	PpgA	F-Box protein, RCC1 domain, activates Ran, affects host cell motility	(125)
lpg2464	DrrA^*b*^	Rab1 guanine nucleotide exchange factor, binds phosphatidylinositol 4-phosphate	(126)
lpg2490	LepB^*b*^	Inactivator Rab1, by acting as a GTPase-activating protein, has phosphatidylinositide 4-kinase activity	(127, 128)
lpg2504	SidI	Inhibition of translation, promotion of cell death, inhibition of UPR	(129)
lpg2862	Lgt2/LegC8^*b*^	Inhibition of elongation factor 1A function, UDP-glucosyltransferase, modifies eEF1A; modulates autophagy	(129, 130)

^*a*^ We selected those effectors that had 50% or more effect on either recRNA-S or recRNA-L accumulation or on both recRNAs.

^*b*^ *Legionella* effectors that affected TBSV replication in yeast.

Analyzing the known host targets of the identified *Legionella* effectors helped group them to several cellular pathways. For example, the targeted cellular factors by the *Legionella* effectors, which were identified in this work, are those that (i) are part of the actin network (targeted by RavK and VipA) (117); (ii) affect the autophagy pathway (LegA9, RavZ, Lgt2/LegC8, and LegS2) (116, 124, 129, 131) or (iii) target the elongation factor 1A (eEF1A) (Lgt2/LegC8) (129). Several identified effectors inhibit Rab1 GTPase function (DrrA and LepB) (126, 127), bind to/modify phosphoinositides, including PI(3)P or PI(4)P (LepB, DrrA and RavZ) (128), or target the endosomal and vesicle trafficking (Ceg19 and LegC3) (109, 121). Several of these host proteins and pathways are co-opted for TBSV replication (see Discussion) (103, 104, 132).

To validate our screening approach with bacterial effectors, we chose the RavK effector that destroys the actin filaments by cleaving Act1 in yeast (117). We have shown previously that the hijacked actin network plays a major role in TBSV recombination by affecting the assembly of the viral replicase complex and subversion of host cellular helicases, such as the DDX3-like Ded1 and eIF4AIII-like RH2 DEAD-box helicases, which affect template switching by the TBSV replicase (47, 59, 60, 133). We found that the expression of RavK effector increased TBSV recRNA accumulation by ~2.5× when compared to control in yeast carrying temperature-sensitive Act1 protein (Fig. 2C). Altogether, the bacteria effector-based screen led to the identification of novel cellular targets and pathways that affect viral RNA recombination, demonstrating that bacterial effectors are useful probes to unravel viral processes in eukaryotic cells.

### Screening of SAR-CoV-2 and HMPV proteins to identify host pathways and host proteins that alter tombusvirus replication and RNA recombination in yeast

**Fig 3:**
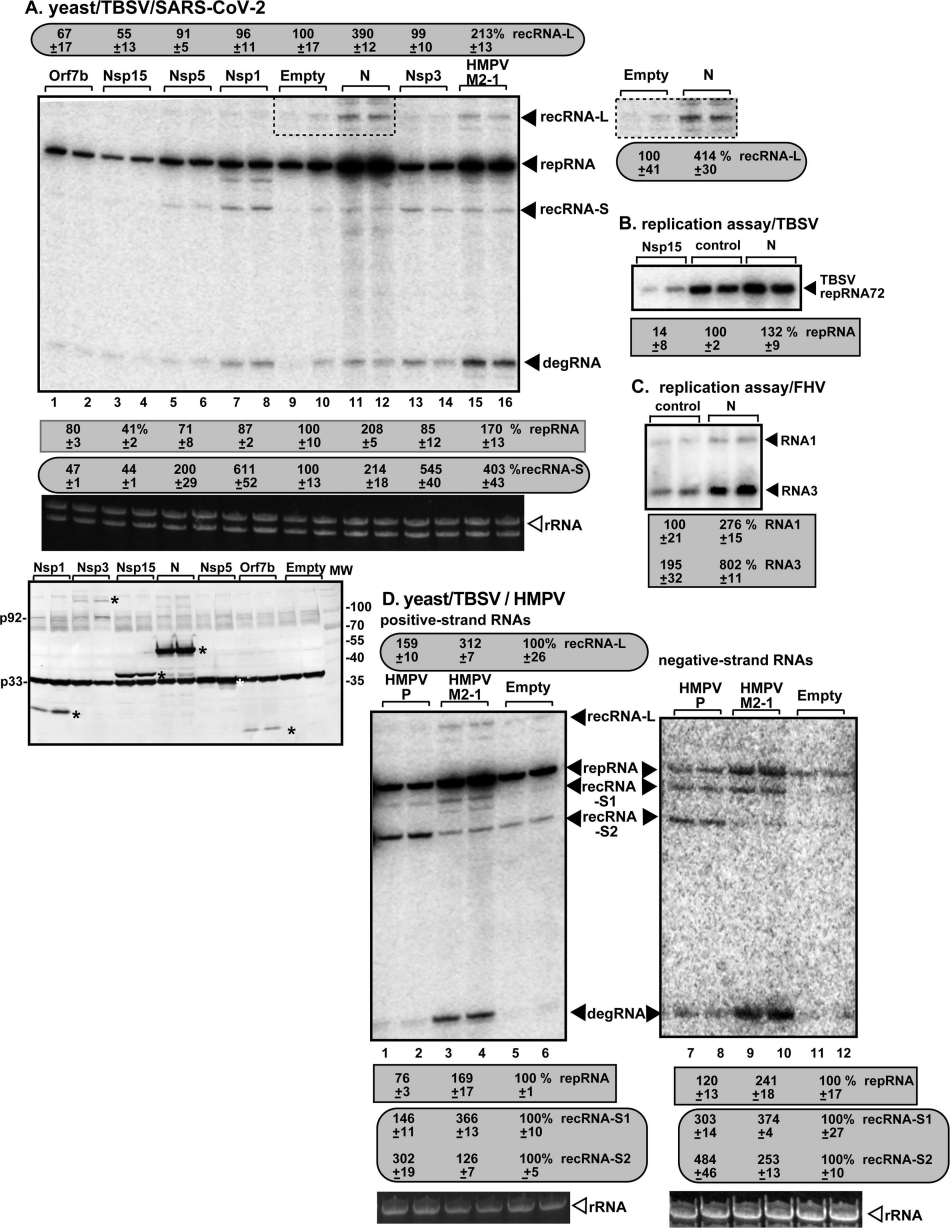
Expression of SARS-CoV-2 and HMPV proteins affects TBSV RNA recombination in yeast. (**A**) The effects of separate expression of SARS-CoV-2 proteins on TBSV repRNA accumulation and recRNA production were measured by northern blot 24 h after initiation of TBSV replication via expression of p33, p92^pol^ replication proteins, and DI-AU-FP repRNA in BY4741 yeast. The accumulation level of repRNA was taken as 100% in yeast not expressing heterologous viral protein. The production of recRNA-L and recRNA-S in yeast expressing the given SARS-CoV-2 proteins was compared to the corresponding recRNAs in WT yeast (100%). Note that the entire boxed area on the right, which includes recRNA-L and an additional longer recRNA, was used to measure recRNA level produced in yeast expressing the SARS-CoV-2 N protein. Each sample is obtained from independent yeast colonies. Bottom image shows western blot of the expression of the given SARS-CoV-2 proteins and the tombusvirus p33 and p92^pol^ replication proteins detected by anti-His antibody in yeast. Each experiment was repeated. (**B**) The accumulation level of repRNA72 in yeast expressing the given SARS-CoV-2 proteins. Note that repRNA72 (DI-72) replicates at a much higher level than DI-AU-FP repRNA, and also, it is less recombinogenic than DI-AU-FP due to missing the AU-FP region (Fig. 1B). (**C**) The accumulation level of FHV RNAs in yeast expressing SARS-CoV-2 N protein. Note that subgenomic RNA3 is produced from the FHV genomic RNA1 by the viral replicase. (**D**) The effects of separate expression of HMPV proteins on TBSV repRNA accumulation and recRNA production were measured by northern blot 24 h after initiation of TBSV replication via expression of p33, p92^pol^ replication proteins, and DI-AU-FP repRNA in BY4741 yeast. The accumulation level of repRNA was taken as 100% in yeast not expressing heterologous viral protein. The production of recRNA-L, recRNA-S1, and the shorter recRNA-S2 in yeast expressing the given HMPV proteins was compared to the corresponding recRNAs in WT yeast, which were taken as 100%. The panel on the right shows the production of negative-strand RNA products. Each experiment was repeated.

Viral proteins expressed by different viruses in host cells might target common host factors that are subverted by TBSV, thus resulting in competition for resources needed by TBSV (Fig. 1A). Alternatively, the heterologous viral proteins could inhibit the function of yeast antiviral (anti-TBSV) proteins, thus affecting TBSV replication or recombination. To test this idea, first, we individually expressed 27 SARS-CoV-2 proteins (134) in yeast also replicating TBSV DI-AU-FP repRNA. Three SARS-CoV-2 proteins were not detectable (Nsp4, Nsp11, and S structural proteins) in yeast. We found that the structural N nucleocapsid protein enhanced TBSV repRNA accumulation by ~2-fold (Fig. 3A, lanes 11–12). Interestingly, the SARS-CoV-2 N protein also enhanced the replication of a low-recombinogenic TBSV repRNA (repRNA72, Fig. 3B). In addition, SARS-CoV-2 N protein stimulated the unrelated insect (+)RNA virus, flock house virus (FHV) genomic RNA1, and subgenomic RNA3 accumulation by ~3- and fourfold, respectively, in yeast (Fig. 3C). Moreover, N, Nsp1, Nsp3, and Nsp5 enhanced TBSV recombination by two- to sixfold (Fig. 3A). The expression of N protein led to ~4-fold increased accumulation of long recRNAs (recRNA-L, Fig. 3A, lanes 11–12, see boxed area). On the contrary, the expression of Nsp15 and Orf7b reduced TBSV recombinant recRNA generation by ~2-fold, close to undetectable level (Fig. 3A, lanes 3–4), while Nsp15 also inhibited replication of the TBSV replicon RNAs by up to ~7-fold (Fig. 3B).

Separate expression of eight of the negative-strand HMPV proteins in yeast replicating TBSV repRNA revealed that the auxiliary phosphoprotein (P) and M2-1 enhanced TBSV RNA recombination by ~3-fold (Fig. 3D). Whereas the HMPV M2-1 protein stimulated TBSV replication by 70%, the P protein had no effect (Fig. 3D). Interestingly, the expression of P and M2-1 enhanced (−)recRNA accumulation by ~3- to 5-fold. The TBSV recombination profile induced by HMPV M2-1 was similar to that observed for the SARS-CoV-2 N protein (compare Fig. 3A lanes 15–16 with 11–12), whereas the TBSV recombination profile induced by HMPV P protein was different. The former ones supported both recRNA-L and recRNA-S types, while the latter one supported mostly recRNA-S2 production (Fig. 3A and D). We previously found that different host factors greatly affect TBSV recombination profiles, which might be useful to identify the targeted host factor(s) (37, 46, 51, 52, 58, 113).

The above-identified SARS-CoV-2 and HMPV proteins affect various cellular processes (Table 2), including autophagy, host translation, membrane shape, RNA stability, and virion assembly. Altogether, the identification of these heterologous viral proteins as modifiers of TBSV replication and recombination suggests common and shared host factors among these heterologous proteins and TBSV proteins, and in general, among these RNA viruses in eukaryotic cells.

**TABLE 2:** Identified SARS-CoV-2 and HMPV viral proteins affecting TBSV RNA recombination in yeast^*a*^

Viral protein	Functions
SARS-CoV-2	
N protein	Virion assembly by interacting with the genomic RNA. N protein inhibits RNAi and dsRNA recognition (135). The N protein forms biocondensates, which are crucial for viral replication through concentrating the viral RNA with the host cell's protein synthesis machinery (136). The N protein is also involved in viral RNA recombination, as shown by the emergence of new recombinants carrying mutations in the N gene (137). N protein induces autophagy by inhibiting mTOR activity via enhancing LARP1 activity (138).
Nsp1	Endonuclease that cleaves within the 5´ UTR of mRNAs, thus inhibiting host translation and redirecting ribosomes to viral protein synthesis (139).
Nsp3	Very large membrane-bound protein with protease activity. Nsp3 is part of the pore-forming complex in the double-membrane VROs (140). Nsp3 enhances viral translation, and its macro domain removes adenosine diphosphate ribosylation, a post-transcriptional protein modification, to dampen hosts’ immune responses (141, 142). Nsp3 also disrupts the autophagy pathway (138).
Nsp5	The main protease, which cleaves the viral polyproteins, releasing the matured proteins. Nsp5 also cleaves host proteins, which affects viral infections (143). Nsp5 targets ATG1/ULK1 kinase and p62 cargo receptor of the autophagy pathway to disrupt autophagy (144–146). The activity of Nsp5 is affected by autophagy through ubiquitination by the host Parkin E3 ligase (147).
Nsp15	A uracil-specific endoribonuclease that preferentially cleaves pyrimidine nucleotides within thermodynamically less stable regions in dsRNAs, such as AU-rich or mismatch-containing areas (140,148).
Orf7b	Small auxiliary protein that is known to interfere with immune signaling (149), and it induces apoptosis (150, 151). Orf7b might act as a viroporin by forming an ion channel across membranes (152) and might disrupt olfactory receptors.
HMPV	
P protein	Phosphoprotein, a co-factor between the N protein and the L protein (RdRp), required for inclusion body formation, and it drives phase separation and liquid-like bodies formation (153). P protein reorganizes the host cytoskeleton.
M2-1	Transcription antiterminator and RNA polymerase processivity factor that prevents premature polymerase termination (154). M2-1 is also involved in virus particle assembly.

^*a*^ UTR, untranslated region.

### SARS-CoV-2 N protein protects TBSV RNAs from Xrn1 5´−3´ exoribonuclease to enhance TBSV replication and recombination in yeast

**Fig 4:**
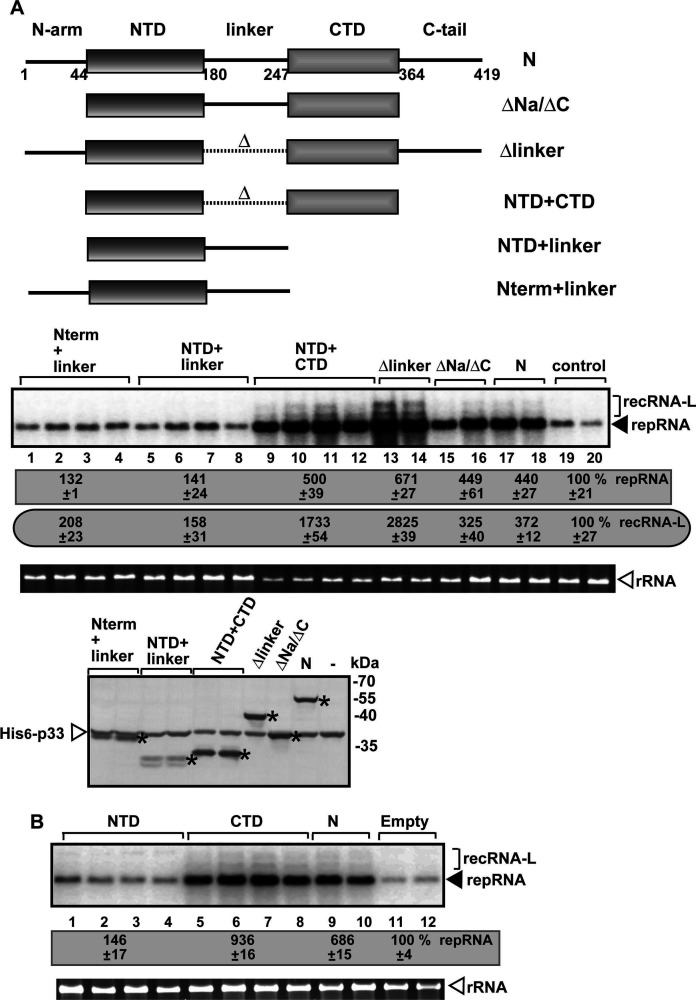
The effects of the expression of various domains of the SARS-CoV-2 N protein on TBSV replication and RNA recombination in yeast. (**A**) Schematic representation of the different domains in the N protein and the expressed mutants. NTD domain facilitates binding to the viral genomic RNA, whereas CTD domain promotes N protein dimerization (135). The effects of separate expression of SARS-CoV-2 N protein mutants on TBSV repRNA accumulation and recRNA-L production were measured by northern blot 24 h after initiation of TBSV replication via expression of p33, p92^pol^ replication proteins, and DI-AU-FP repRNA in BY4741 yeast. See further details in Fig. 3A legend. Bottom image shows western blot of the expression of the SARS-CoV-2 N protein mutants and the p33 replication protein detected by anti-His antibody in yeast. Each experiment was repeated. (**B**) The effect of separate expression of HA-tagged NTD and CTD domains of the SARS-CoV-2 N protein on TBSV repRNA replication in yeast. See further details in panel A.

To validate our screening approach and uncover how the SARS-CoV-2 N protein enhances TBSV RNA replication and recombination in yeast, first, we have characterized the domains of the N protein involved in stimulating TBSV replication and RNA recombination. Interestingly, the expression of the combined NTD and CTD domains, in the absence of the linker sequence, stimulated TBSV repRNA accumulation by ~5- to sevenfold and RNA recombination by ~17- to 28-fold (Fig. 4A, lanes 9–14) in yeast. This is a much higher level of TBSV replication and recombination stimulation than that caused by the expression of the full-length N protein (Fig. 4A, lanes 17–18). Expression of the CTD domain alone enhanced TBSV repRNA accumulation and recRNA production (Fig. 4B), suggesting that the CTD protein interaction domain is more important than the NTD nucleotide-binding domain in affecting TBSV replication. Altogether, these data suggest that the SARS-CoV-2 N protein has the potential to affect replication and RNA recombination of other viruses.

**Fig 5:**
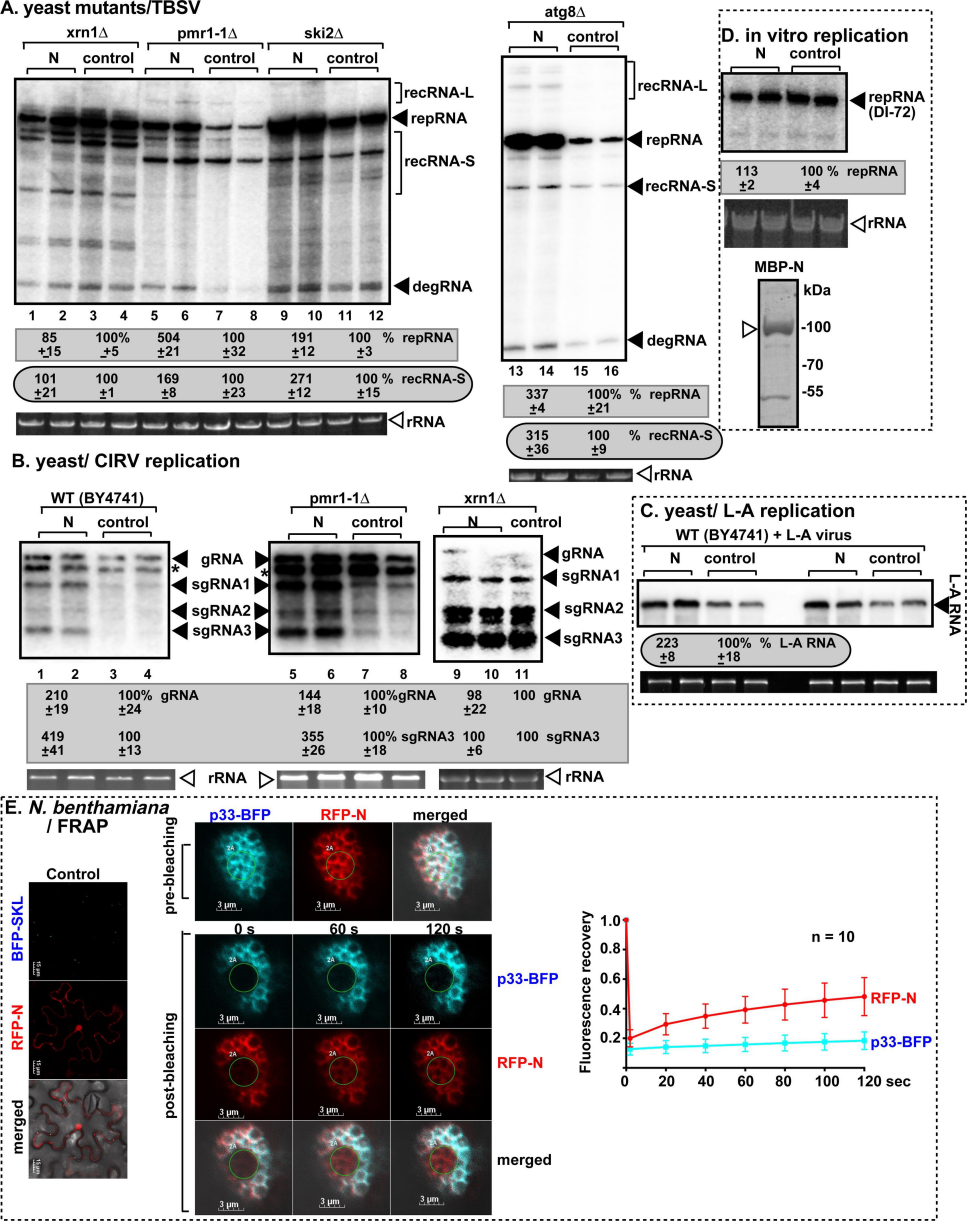
Effects of expression of SARS-CoV-2 on TBSV replication and RNA recombination in yeast strains. (**A**) The effects of expression of SARS-CoV-2 N protein on TBSV repRNA accumulation and recRNA production were measured by northern blot 24 h after initiation of TBSV replication via expression of p33, p92^pol^ replication proteins, and DI-AU-FP repRNA in xrn1Δ, pmr1-1Δ, ski2Δ, or atg8Δ yeast strains. The accumulation level of repRNA was taken as 100% in yeast strains not expressing the N protein. The production of recRNA-S in yeast expressing the SARS-CoV-2 N protein was compared to the corresponding recRNAs produced in control yeast, which were taken as 100%. (**B**) The effect of expression of SARS-CoV-2 N protein on CIRV genomic gRNA and sgRNA3 accumulation was measured by northern blot 24 h after initiation of CIRV replication in xrn1Δ, pmr1-1Δ, and WT yeast strains. The accumulation levels of gRNA and sgRNA3, respectively, were taken as 100% in yeast strains not expressing the N protein. Asterisk indicates a short CIRV genomic RNA lacking 5´ end sequences. (**C**) The effects of expression of SARS-CoV-2 N protein on yeast L-A virus RNA were measured by northern blot 24 h after N expression in WT yeast strain carrying L-A virus. (**D**) *In vitro* replication assay does not support a direct role for the N protein in TBSV replicase assembly and function. Membrane-enriched fraction from WT yeast was programmed with *Escherichia coli*-expressed purified TBSV p92 and p33 replication proteins and TBSV DI-72 (+)repRNA in the presence of purified MBP-N protein or MBP *in vitro*. Denaturing PAGE analysis of the ^32^P-labeled TBSV RNA products obtained is shown. (**E**) Fluorescence recovery after photobleaching (FRAP) analysis shows partial fluorescence recovery of red fluorescent protein (RFP)-N protein after photobleaching in a single VRO induced by TBSV p33-BFP in *Nicotiana benthamiana*. Left panel: localization of RFP-N protein in the cytosol and possibly in the nucleus of *N. benthamiana* cell. Scale bars represent 15 µm. Center, top panel: co-localization of replication protein p33-BFP and RFP-N protein in a single VRO before photobleaching. Bottom panels: confocal microscopy images show partial fluorescence recovery of RFP-N protein at 0, 60, and 120 seconds post-photobleaching within a VRO. The photo-bleached area is circled. Note that signals of p33-BFP did not recover in the FRAP assay because it is a membrane-bound protein. Scale bars represent 3 µm. Right panel: the graph shows time course analysis of FRAP data on RFP-N protein signal recovery in individual VROs. Sample size *n* is annotated in the figure. Each experiment was repeated.

To identify the cellular target of SARS-CoV-2 N protein that is critical for its effect on TBSV, we used yeast strains deficient in previously defined cellular functions affecting TBSV replication or recombination. These included yeast mutants deficient in (i) 5´-to-3´ RNA degradation (xrn1Δ), (ii) 3´-to-5´ RNA degradation (ski2Δ), (iii) Mn^++^/Ca^++^ intracellular transport between the cytosol and Golgi (pmr1-1Δ, which affects Pmr1p Mn^++^/Ca^++^ pump function) (46), or (iv) autophagy (atg8Δ). We observed that the expression of N protein enhanced both replication (two- to fivefold) and RNA recombination (~2- to threefold) of TBSV in ski2Δ, pmr1-1Δ, and atg8Δ yeast strains (Fig. 5A) when compared with WT yeast (Fig. 3A). These data suggest that the above yeast proteins did not have a major effect on N protein stimulatory function in TBSV replication/recombination in yeast. However, deletion of Xrn1 5´−3´ exoribonuclease in yeast resulted in similar TBSV replication and recombination levels in the presence of N protein to that observed in its absence (Fig. 5A, lanes 1–2 versus 3–4). This finding provides evidence that the expression of SARS-CoV-2 N protein leads to the protection of TBSV RNAs from Xrn1 5´−3´ exoribonuclease function in TBSV replication and recombination. This observation was further supported by the enhanced accumulation of full-length genomic (g)RNA of carnation Italian ringspot tombusvirus (CIRV), including the production of subgenomic sgRNA1/2 and sgRNA3, in both WT and pmr1-1Δ yeast expressing the SARS-CoV-2 N protein (Fig. 5B). CIRV genomic RNA replicates in yeast and produces subgenomic RNAs (Fig. 5B), including sgRNA1/2 and sgRNA3. As expected, the CIRV gRNA is more resistant to Xrn1 5´−3´ exoribonuclease due to its unique 5´ untranslated region (UTR) structure than the sgRNAs (155), and the N protein protected the Xrn1-sensitive sgRNAs more than the gRNA (Fig. 5B). This supports the notion that SARS-CoV-2 N protein may lead to protection of the Xrn1-sensitive CIRV sgRNAs in yeast. To further demonstrate that SARS-CoV-2 N protein affects Xrn1 antiviral activity, we tested the replication of L-A dsRNA virus of yeast, which is sensitive to Xrn1 activity (156). Expression of SARS-CoV-2 N protein in yeast infected with L-A virus resulted in over twofold increased L-A virus RNA accumulation (Fig. 5C). TBSV and yeast L-A virus share Xrn1 as a common antiviral factor based on previous genomic screens in yeast (49, 156, 157), supporting our model that N protein prevents Xrn1 antiviral activity in yeast.

The purified N protein did not affect TBSV replication in *in vitro* replicase assembly assay (Fig. 5D), suggesting that the N protein does not directly affect TBSV replicase function. Interestingly, confocal imaging revealed that SARS-CoV-2 N protein accumulated in TBSV VROs in *Nicotiana benthamiana* cells (Fig. 5E). Fluorescence recovery after photobleaching (FRAP) analysis revealed that, unlike for the membrane-bound p33-BFP, the signals for red fluoresecent protein (RFP)-N were partially recovered (Fig. 5E). This suggests that the N protein is present in TBSV p33 replication protein-induced vir-condensate associated with the membranous VRO. Partitioning of N protein in TBSV vir-condensates is likely due to its multivalent interactions with host proteins recruited/shared with TBSV p33.

We suggest that the SARS-CoV-2 N protein enhances TBSV replication, RNA recombination, and CIRV subgenomic RNA production by preventing the degradation of TBSV and CIRV RNAs by Xrn1 ribonuclease, which is known to be a major host factor suppressing TBSV replication and recombination in yeast and plants (38, 49, 51, 52, 113). Altogether, this is a novel function for the SARS-CoV-2 N protein, illustrating that the TBSV/yeast system could be used as a system sensor to gain novel discoveries on functions of heterologous pathogen/virus proteins.

### Enhancement of TBSV replication and recombination in yeast by HMPV M2-1 protein depends on inhibition of antiviral function of Xrn1 5´−3´ ribonuclease

**Fig 6:**
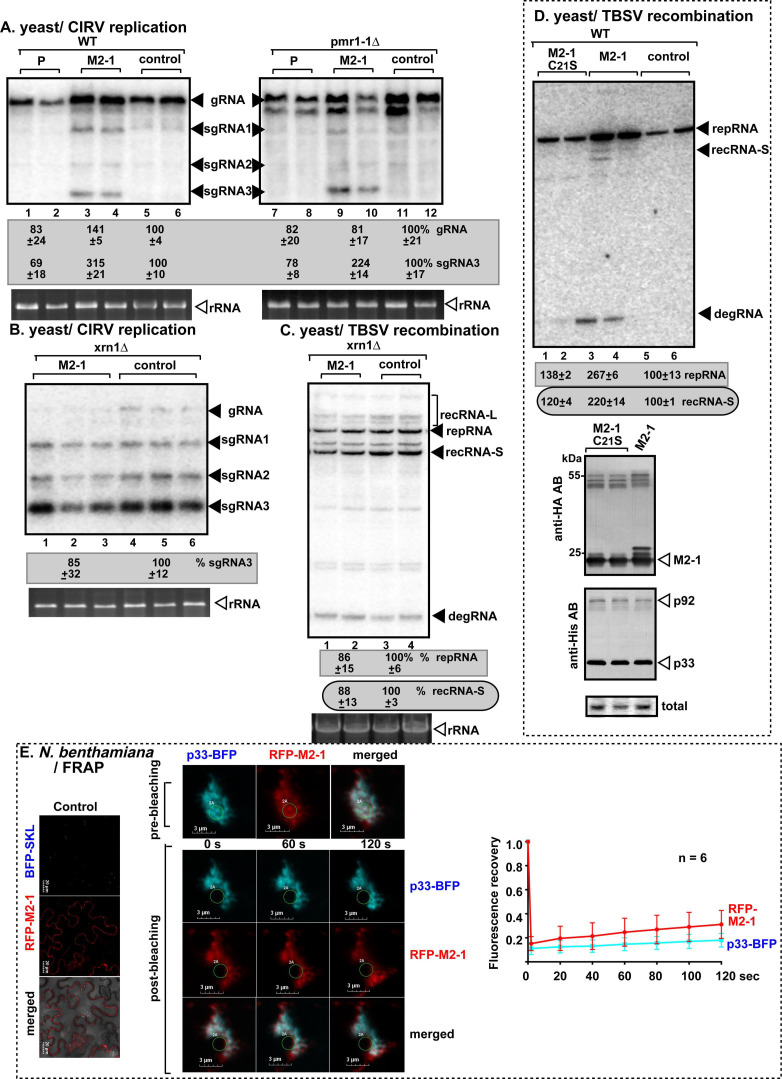
Effects of expression of HMPV proteins on TBSV replication and RNA recombination in yeast strains. (**A, B**) The effects of expression of HMPV M2-1 protein on CIRV gRNA and sgRNA3 accumulation were measured by northern blot 24 h after initiation of CIRV replication in WT, pmr1-1Δ, and xrn1Δ yeast strains. The accumulation levels of gRNA and sgRNA3, respectively, were taken as 100% in yeast strains not expressing the M2-1 or P proteins. (**C**) The effects of expression of HMPV M2-1 protein on TBSV repRNA accumulation and recRNA production were measured by northern blot 24 h after initiation of TBSV replication via expression of p33, p92^pol^ replication proteins, and DI-AU-FP repRNA in xrn1Δ yeast strain. The accumulation levels of repRNA and recRNAs, respectively, were taken as 100% in yeast strains not expressing the M2-1 protein. (**D**) The effects of expression of HMPV M2-1 protein (lanes 3–4) and the zinc-binding mutant M2-1 protein (lanes 1–2) on TBSV repRNA accumulation and recRNA production were measured by northern blot 24 h after initiation of TBSV replication via expression of p33, p92^pol^ replication proteins, and DI-AU-FP repRNA in WT yeast strain. Bottom images show western blots to detect the accumulation of HMPV M2-1 and TBSV p33 and p92 replication proteins detected with anti-HA and anti-His antibodies, respectively. See further details in panel C. (**E**) FRAP analysis shows partial fluorescence recovery of RFP-M2-1 protein after photobleaching in a single VRO induced by TBSV p33-BFP in *N. benthamiana*. See further details in Fig. 5E. Each experiment was repeated.

To identify the shared host factor targeted by HMPV M2-1 and P proteins and TBSV, we compared the TBSV RNA recombination profiles produced by the SARS-CoV-2 and HMPV proteins. Interestingly, we observed that the expression of HMPV M2-1 and SARS-CoV-2 N proteins resulted in similar TBSV RNA recombination profiles by producing both recRNA-L and recRNA-S products (compare Fig. 3A, lanes 11–12 versus 15–16), whereas HMPV P protein-induced TBSV RNA recombination profile was different (compare Fig. 3D, lanes 1–2 versus 3–4). Further testing of HMPV M2-1 with yeast mutants replicating CIRV genomic RNA revealed that sgRNA3 was more stable/accumulated to a higher level in WT and pmr1-1Δ yeast expressing M2-1 protein than in yeast expressing P protein or in control yeast not expressing HMPV proteins (Fig. 6A). However, CIRV sgRNA3 was as stable in xrn1Δ yeast as in WT yeast expressing M2-1 or not expressing HMPV proteins (compare Fig. 6B, lanes 1–3 versus 4–6). In another test, the TBSV RNA recombination profile was comparable in xrn1Δ yeast expressing HMPV M2-1 or not expressing HMPV proteins (compare Fig. 6C, lanes 1–2 versus 3–4). All these data support the notion that HMPV M2-1 protein blocks the antiviral function of Xrn1 5´−3´ exoribonuclease in yeast, thus affecting TBSV and CIRV replication and RNA recombination. Mutagenesis of the zinc-binding domain of M2-1 involved in RNA-binding (158, 159) rendered the mutant ineffective in stimulating TBSV repRNA replication and RNA recombinant accumulation (Fig. 6D, lanes 1–2 versus 3–4). This suggests that the zinc-binding region could be involved in the regulation of host Xrn1 ribonuclease activity.

Confocal imaging revealed that HMPV M2-1 protein colocalized with TBSV p33 replication protein in TBSV VROs in *N. benthamiana* cells (Fig. 6E). In the FRAP-based assay, the signal for RFP-M2-1 was partially recovered (Fig. 6E), suggesting that the partitioning of HMPV M2-1 in TBSV vir-condensates is likely due to weak multivalent interaction of HMPV M2-1 with host proteins that are recruited/shared with TBSV p33. Altogether, the determination of a novel function for the HMPV M2-1 protein in the regulation of host Xrn1 activities further illustrates that the TBSV/yeast system could be used as a system sensor to identify novel functional targets of heterologous viral proteins.

### Role of the autophagy pathway in TBSV RNA recombination

Because four *Legionella* effectors (Table 1) and three SARS-CoV-2 proteins (Table 2) identified in the above TBSV recombination screens are predicted/known to affect/modulate the autophagy pathway and autophagy is known to function to facilitate TBSV replication (99), we decided to further characterize its role in TBSV RNA recombination.

**Fig 7:**
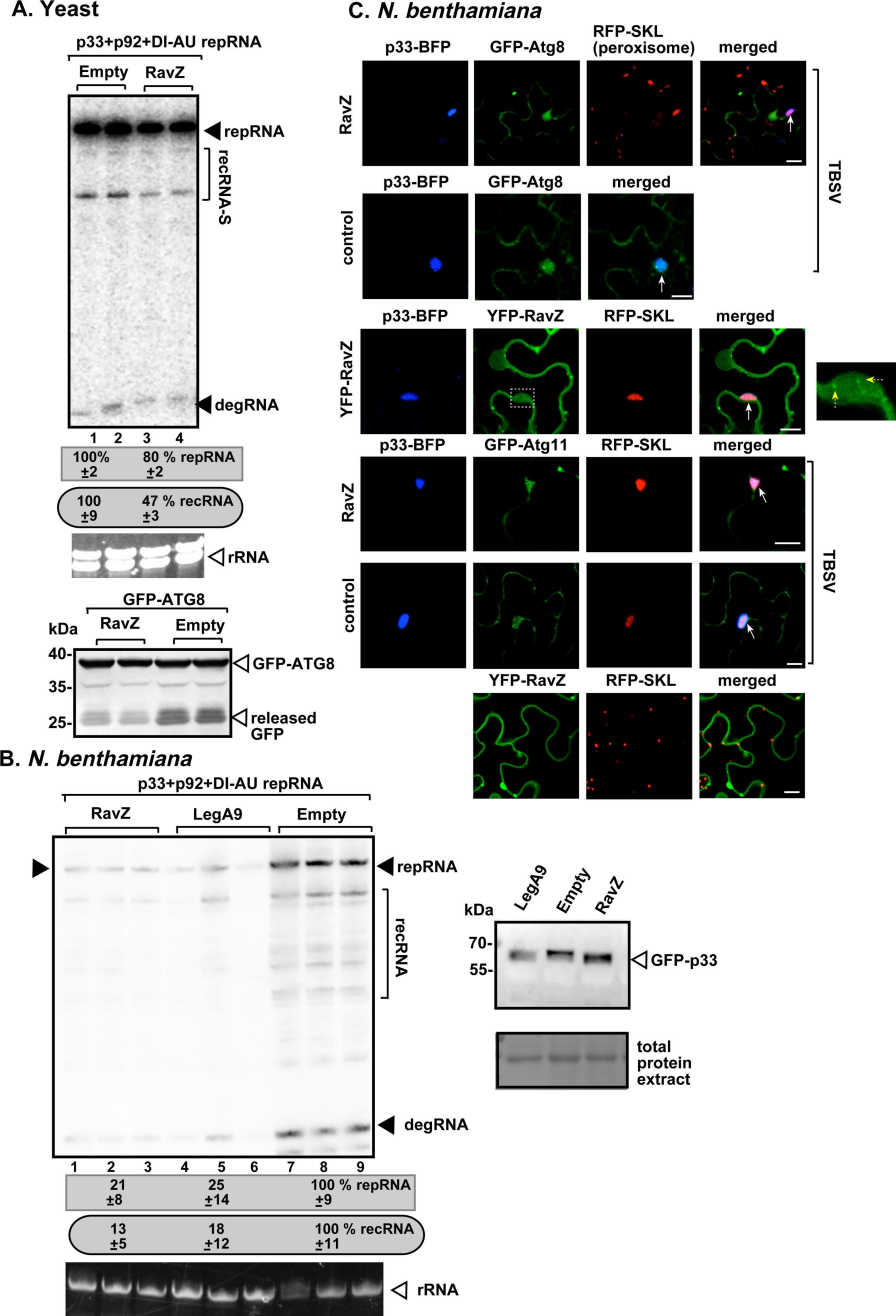
Expression of the *Legionella* RavZ effector inhibits TBSV replication and RNA recombination in yeast and plants. (**A**) The effects of *Legionella* RavZ effector on TBSV repRNA accumulation and recRNA production were tested in BY4741 yeast. Accumulation of TBSV DI-AU-FP repRNA and recRNA-S was measured by northern blot 36 h after initiation of TBSV replication in yeast. The accumulation levels of repRNA and recRNA-S were taken as 100% in yeast not expressing effectors. Each sample was obtained from independent yeast colonies. Bottom panel shows the release of “free GFP” from GFP-ATG8 in yeast expressing RavZ or not expressing RavZ in control. (**B**) Inhibition of TBSV replication and recombination in *N. benthamiana* by RavZ and LegA9 expressions. Total RNAs were extracted from leaves 1.5 days after agroinfiltration to express TBSV p33, p92, and the DI-AU-FP repRNA. Note that the same leaves were agroinfiltrated 2.5 days earlier to express RavZ or LegA9 effectors or no effectors (as a control). Northern blot analysis was performed on total RNA extracts obtained from separate leaf samples. The accumulation level of repRNA and recRNA-S was calculated as described in panel A. Panel on the right shows western blot analysis of the accumulation of TBSV GFP-p33 replication protein, detected by anti-GFP antibody, in plant cells expressing RavZ or LegA9 effectors or no effectors (as a control). Each experiment was performed three times. (**C**) Top two panels: confocal laser microscopy analysis of plant cells expressing p33-BFP replication protein, GFP-ATG8 autophagy core protein, RFP-SKL peroxisomal luminar marker, and untagged RavZ or no *Legionella* effector (as a control). The leaves were also inoculated with TBSV. The VROs formed in plant cells are marked with arrows. Third panel: similar analysis except YFP-RavZ was expressed in plant cells. Note that the enlarged image on the right shows that YFP-RavZ formed small punctate structures (marked with yellow arrows) within the large VROs. Bottom panels: confocal laser microscopy analysis of plant cells expressing proteins as above, except GFP-ATG11 autophagy scaffold protein was also expressed as shown. Scale bars represent 10 µm. See further details above. Each experiment was repeated.

Ectopic expression of *Legionella* RavZ reduced the accumulation of (+)recRNAs by ~50% and ~90% in yeast and *N. benthamiana* leaves, respectively, (Fig. 7A and B), suggesting that inhibition of autophagy leads to reduced RNA recombination. RavZ is known to cleave PE-lipidated ATG8 autophagy core protein and bind to PI(3)P-rich highly curved membranes, such as the autophagosome (119, 160). We showed that RavZ cleaves green fluorescent protein (GFP)-ATG8 (99) and, by inhibition of autophagy, results in reduced release of free GFP from GFP-ATG8 in yeast cells (Fig. 7A, bottom image). However, the expression of RavZ did not affect the level of GFP-p33 in plant cells (Fig. 7B), demonstrating that p33 replication protein is unlikely the target of RavZ. Confocal microscopy-based analysis revealed that RavZ expression interfered with the robust recruitment of GFP-ATG8 to the TBSV VROs formed by the p33-BFP replication protein in clustered peroxisomal membranes (Fig. 7C). This is in contrast with the robust recruitment of GFP-ATG8 into VROs in control plant cells replicating TBSV (Fig. 7C) (99). Note that in control plant cells, GFP-ATG8 forms several puncta, which represent condensate substructures, called vir-NBR1 bodies, associated with the membranous VROs as we have shown previously (75, 99). Interestingly, YFP-RavZ is partly colocalized with TBSV VROs (Fig. 7C, third panel), likely due to the presence of either co-opted ATG8 in VROs or the highly curved PI(3)P-enriched membranes within VROs (99, 161–163). Moreover, the expression of RavZ did not affect the recruitment of ATG11 autophagy scaffold protein to VROs (Fig. 7C), which has pro-viral function (164). Thus, RavZ seems to target ATG8, but not all autophagy components, within the VRO. Nevertheless, our data show that RavZ expression, thus inhibition of autophagy, reduces TBSV RNA recombination in yeast and plants.

**Fig 8:**
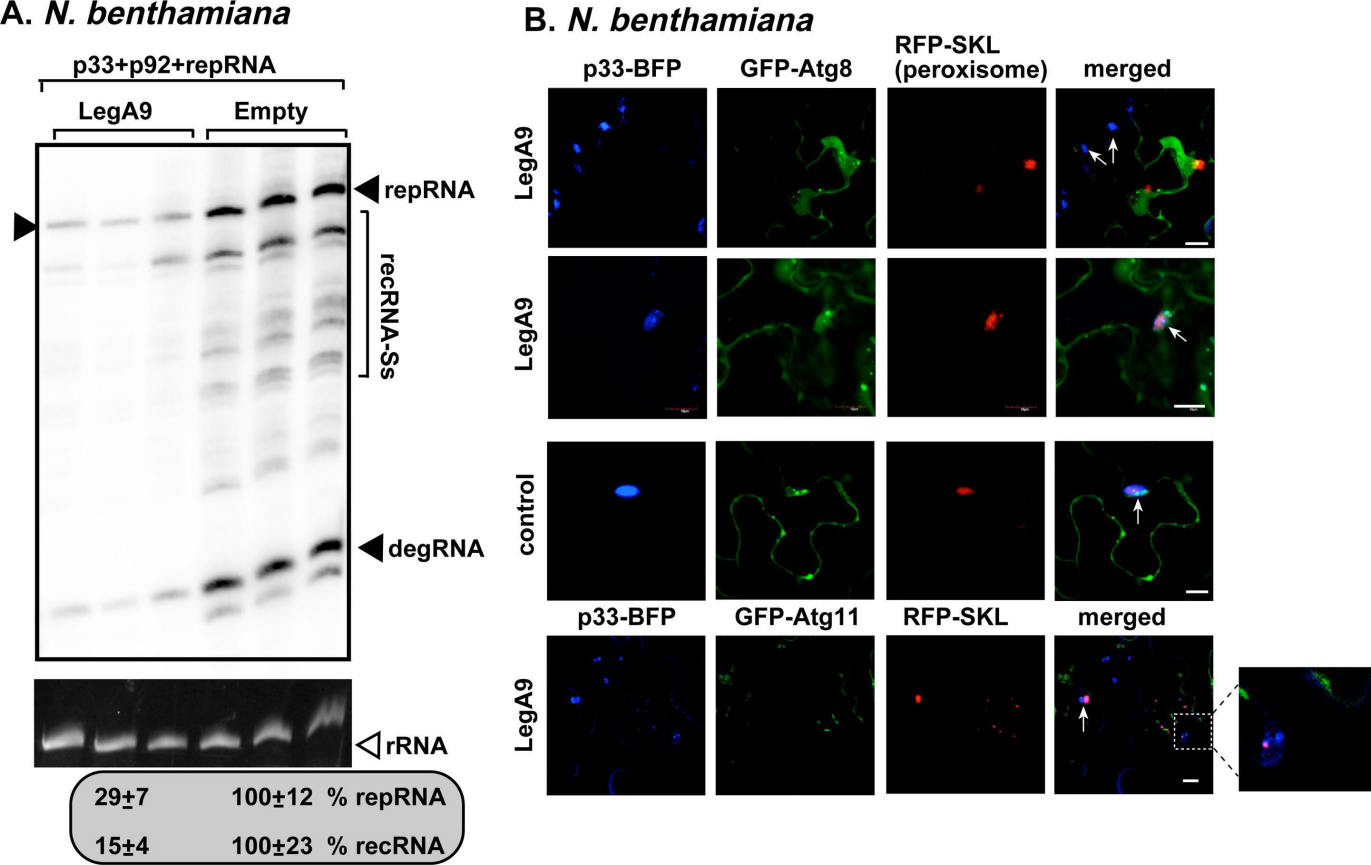
Expression of the *Legionella* LegA9 effector inhibits TBSV replication and RNA recombination in yeast and plants. (**A**) Inhibition of TBSV replication and recombination in *N. benthamiana* by LegA9 expression. Total RNAs were extracted from the leaves 1.5 days after co-agroinfiltration to express TBSV p33, p92, the DI-AU-FP repRNA, and LegA9 effector or no effector (as a control as described in Fig. 7B), followed by northern blot analysis using separate leaf samples. The accumulation level of repRNA and recRNA-S was calculated as described in panel A. Each experiment was performed three times. (**B**) Top three panels: confocal laser microscopy analysis of plant cells expressing p33-BFP replication protein, GFP-ATG8 autophagy core protein, RFP-SKL peroxisomal luminar marker, and untagged LegA9 or no *Legionella* effector (as a control, third panel). The leaves were also inoculated with TBSV. The VROs formed in plant cells are marked with arrows. Fourth panel: similar analysis except GFP-ATG11 autophagy scaffold protein was expressed as shown. Scale bars represent 10 µm. See further details above. Note that the enlarged image on the right shows that GFP-ATG11 did not colocalize with the VRO. Each experiment was repeated.

To further test the effect of autophagy on TBSV recombination, we exploited the *Legionella* LegA9 effector, which is known to inhibit autophagy (116), and it was identified in our screen (Table 1). Expression of the LegA9 effector reduced the accumulation of (+)recRNAs in both yeast cells (Fig. 2A, lanes 5–6) and *N. benthamiana* leaves (Fig. 8A), suggesting that inhibition of autophagy leads to reduced RNA recombination. In addition, LegA9 also reduced TBSV replication in both yeast (Fig. 2A, lanes 5–6) and in plants (Fig. 7B, lanes 4–6; and Fig. 8A). Confocal microscopy-based analysis revealed that LegA9 expression interfered with the robust recruitment of GFP-ATG8f and GFP-ATG11 to the TBSV VROs (Fig. 8B). In addition, LegA9 also interfered with the localization of p33-BFP replication protein to peroxisomal membranes in some but not all plant cells (Fig. 8B, compare top and second panels). Altogether, we suggest that LegA9 expression inhibits TBSV replication and RNA recombination by inhibition of the autophagy pathway.

**Fig 9:**
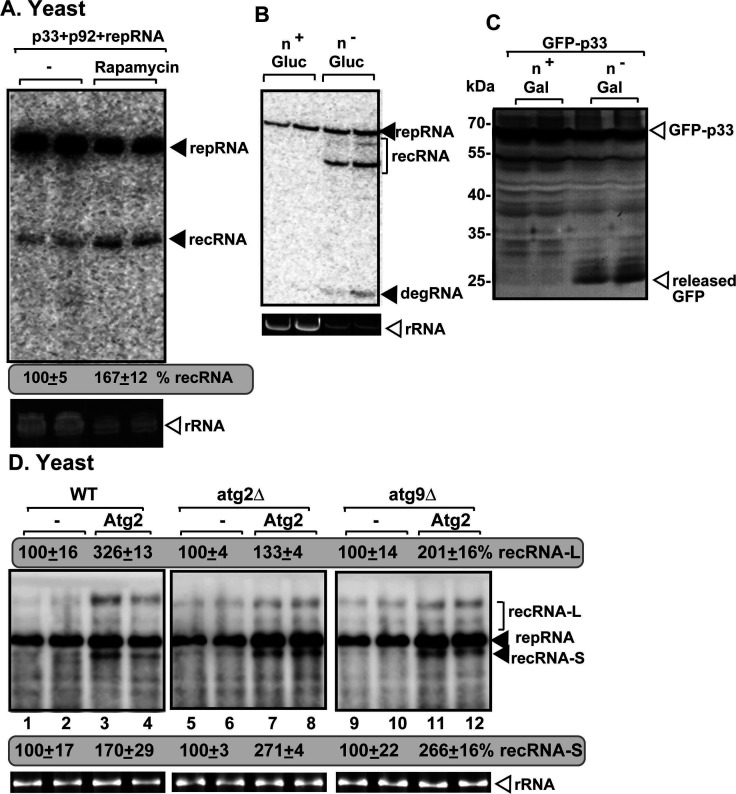
Induction of autophagy stimulates TBSV RNA recombination in yeast. (**A**) The effect of autophagy induction by rapamycin treatment of BY4741 yeast on TBSV recRNA production was measured by northern blot 24 h after initiation of TBSV replication via expression of p33, p92^pol^ replication proteins, and DI-AU-FP repRNA. The accumulation level of recRNA-S was taken as 100% in yeast not treated with rapamycin. Each sample was obtained from independent yeast colonies. Each experiment was repeated. (**B**) The effect of autophagy induction by nitrogen starvation (n^-^) of BY4741 yeast on TBSV recRNA production was measured by northern blot 24 h after initiation of TBSV replication via expression of p33, p92^pol^ replication proteins, and DI-AU-FP repRNA. Note that ribosomal RNAs are present in low amounts when yeast strains are grown under nitrogen starvation. Therefore, we adjusted the samples to have comparable amounts of replicon RNAs for RNA blot analysis. Each sample was obtained from independent yeast colonies. (**C**) Induction of autophagy by nitrogen starvation (n^-^) of BY4741 yeast leads to limited degradation of GFP-p33 as indicated by the release of “free GFP” from GFP-p33 in yeast grown on media lacking nitrogen source. (**D**) Expression of ATG2 bulk phospholipid transfer protein enhances TBSV RNA recombination in yeast. recRNA production was measured by northern blot 24 h after initiation of TBSV replication via expression of p33, p92^pol^ replication proteins, and DI-AU-FP repRNA in WT, atg2Δ, or atg9Δ yeast strains using 1% agarose gel. The productions of recRNA-L and recRNA-S in yeast not expressing ATG2 protein were taken as 100%. Each sample was obtained from independent yeast colonies. Note that the exposure time for the blot in the middle was longer, because ATG2 deletion reduces TBSV repRNA replication in yeast. Each experiment was repeated.

To test if the induction of the autophagy pathway affects TBSV recombination, we treated yeast cells with rapamycin, which is known to inhibit mTOR kinase activity, thus triggering autophagy (165). Although TBSV repRNA replication was reduced by rapamycin treatment (as we have shown previously) (166), TBSV recRNA accumulation was enhanced by ~70% (Fig. 9A). Similarly, nitrogen starvation of yeast cells, which induces robust autophagy (165), led to dramatic enhancement of TBSV recRNA production (Fig. 9B, lanes 3–4). Interestingly, nitrogen starvation led only to modest degradation of p33 replication protein by autophagy in yeast (Fig. 9C), likely due to subversion of the autophagy pathway by TBSV (99). Altogether, the above data suggest that the induction of the autophagy pathway, in general, leads to increased TBSV RNA recombination.

Among the autophagy factors, we have previously found that ATG2 bridge-like bulk phospholipid transfer protein had critical functions in TBSV VRO biogenesis (101). Overexpression of ATG2 led to enhanced TBSV recRNA accumulation in atg2Δ, atg9Δ, and WT yeast strains (Fig. 9D). This suggests that bulk phospholipid transfer by ATG2 lipid transfer protein from the ER to the VROs promotes recombinant RNA formation in yeast. Bulk lipid transfer by the over-expressed ATG2 into the cytosolic leaflet of the acceptor membrane (VRO membrane) might create membrane phospholipid asymmetry and membrane irregularity (167), which could favor RNA recombination via incorrect assembly of VRCs. Altogether, these findings highlight the possibility that autophagy affects TBSV RNA recombination via modulating the phospholipid compositions of VRO membranes.

### Role of phospholipids in TBSV RNA recombination *in vitro*

Autophagosomal membranes are enriched in PE, and phosphatidylcholine (PC) and the minor signaling PI(3)P phospholipid, but poor in protein content (168–170), thus making it suitable for the assembly of new viral replicase complexes. ATG2 bulk phospholipid transfer protein is the major driver of membrane expansion of the autophagosomal membranes (171). ATG2 is also critical to enrich PE, phosphatidylserine (PS), and PI(3)P, the latter via the co-opted Vps34 PI3K complex and PI transfer, within the VROs (101). Albeit TBSV replication leads to higher PE content in yeast cells and *N. benthamiana* leaves (172–174), the actual lipid composition of TBSV VROs is not known.

**Fig 10:**
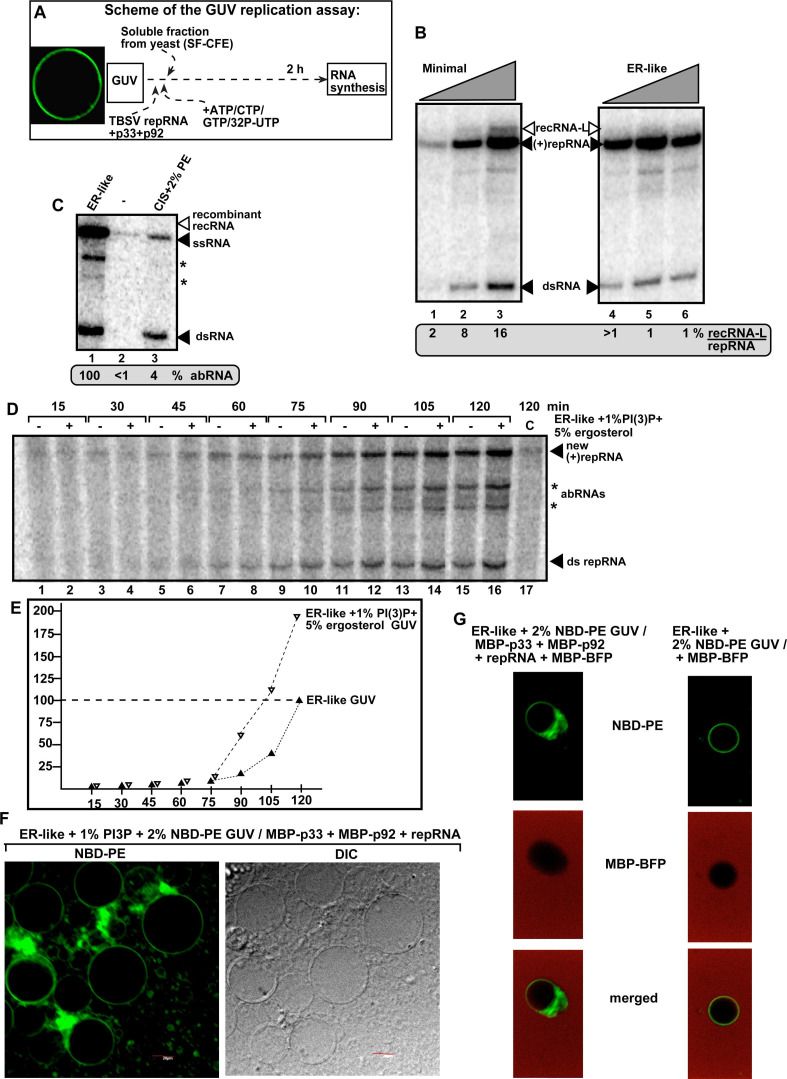
*In vitro* reconstitution of the TBSV replicase in GUVs supports the role of phospholipid composition in TBSV RNA recombination. (**A**) Scheme of the *in vitro* GUV-based TBSV replicase reconstitution assay. Purified recombinant TBSV p33 and p92^pol^ replication proteins in combination with the TBSV (+)repRNA72 (DI-72) were added to different GUV preparations. The S40 fraction of yeast CFE (SF-CFE) was also added to each sample to provide soluble host factors required for TBSV replicase assembly. (**B**) Nondenaturing PAGE analysis of the ^32^P-labeled repRNA and recRNA products obtained is shown. The full-length (+)repRNA progeny, the dsRNA replication intermediate, and the recRNA products are pointed at by arrowheads. The level of recRNA produced in the GUV-based replication assay was compared to the (+)repRNA, which was taken as 100% in each sample. Note that the same amounts of SF-CFE, TBSV p33 and p92^pol^ (200 ng, each), and (+)repRNA (0.25 µg) were used in each reaction, whereas 0.5, 1.0, and 2.0 µL GUVs with “minimal” phospholipid composition (45% PC, 30% PE, 25% PS) (lanes 1–3) or with “ER-like” lipid composition (46% PC, 30% PE, 13% PI, 5% PG, 3% PS, 3% CA) (lanes 4–6) were added to the reaction assays. Each experiment was repeated two times. (**C**) The *in vitro* GUV-based TBSV replicase reconstitution assay was similar to that described in panel B. Note that 2.0 µL GUVs with “*CIS*” phospholipid composition (66% PC, 22% PI, 12% PS + 2% 1,2-dioleoyl-sn-glycero-3 phosphoethanolamine-N-(7-nitro-2–1,3-benzoxadiazol-4-yl) [NBD-PE]) (lane 3) or with “ER-like” lipid composition (lane 1, see in panel B) were added to the reaction assay. Note that no GUV prep was used in the assay as a control (lane 2). Note that recRNAs were not detected under these conditions. Asterisks mark aberrant repRNA products. See further details in panel B. (**D**) The effect of PI(3)P and ergosterol on TBSV repRNA replication. The *in vitro* GUV-based TBSV replicase reconstitution assay was similar to that described in panel B. Note that 2.0 µL GUVs with ER-like lipid composition were used (indicated as “−“) or supplemented with 5% ergosterol and 1% PI(3)P (indicated as “+“) in the time-course experiment. Each sample contained the same amounts of SF-CFE prepared from WT yeast and the purified TBSV p33 and p92 and (+)repRNA72. See further details in panel B. Note that short aberrant RNAs (marked with asterisks), but not the long recRNAs, were detected under these conditions. See further details in panel B. (**E**) The graph shows the relative accumulation of aberrant RNAs in the GUV-based TBSV replicase reconstitution assay shown in panel D. (**F**) Control confocal laser microscopy image of GUVs with purified p33 and p92^pol^ replication proteins and the (+)repRNA72 and SF-CFE. The fluorescent NBD-PE (2%, green) was added to the GUVs with ER-like lipid composition plus 1% PI(3)P supplement. Note the clustering of GUVs and enrichment of PE-containing deformed membranes caused by the TBSV replication proteins. DIC image on the right shows the GUVs. Scale bars represent 20 µm. (**G**) GUVs with ER-like lipid composition + 2% NBD-PE in the presence of p33 and p92^pol^ replication proteins and repRNA72, plus SF-CFE (left panel) or without TBSV components (right panel) were visualized through confocal laser microscopy. Note that purified soluble MBP-BFP was also added to the assay to visualize the lack of soluble components in GUV and the associated PE-containing deformed membranes (left panel). Each experiment was repeated.

To test the roles of phospholipids in TBSV recombination, we used our previously developed GUV-based TBSV replicase assembly assay (175). The replicase prepared with GUVs supports full TBSV repRNA replication when the required phospholipids, for example, with ER-like phospholipid content, were used for GUV preparation (175). Because ATG2 transfers PE, PC, and PS efficiently to autophagosomal membranes, we prepared GUVs with the combination of PE (30%), PC (45%), and PS (25%) (called minimal GUV). This was followed by *in vitro* assembly of the TBSV replicase using the GUV preparation in combination with purified recombinant MBP-p33, MBP-p92, TBSV repRNA, and the soluble cytosolic fraction from WT yeast (soluble fraction of cell-free extract [SF-CFE]) to provide host factors (Fig. 10A) (175). We found that the TBSV replicase activity increased when larger amounts of minimal GUVs were used versus the same amounts of p33/p92^pol^ and (+)repRNA and SF-CFE in the assay (Fig. 10B, lanes 1–3). This was not the case when the more complex ER-like lipid composition was used for GUV preparation (Fig. 10B, lanes 4–6). Importantly, the minimal GUV (when used in the largest amount) supported the production of TBSV recRNAs (Fig. 10B, lane 3), whereas the ER-like GUV produced low levels of recRNAs (Fig. 10, lanes 4–6). These data suggest that both the phospholipid composition of membranes and the protein:phospholipid ratio are factors during TBSV replication and RNA recombination *in vitro*.

Because PE is very critical for TBSV replication in yeast and plants (132, 172) and PE is enriched in TBSV VROs via subversion of ATG2 and the autophagy pathway (99, 101), we tested the role of PE in TBSV recombination. We prepared GUVs with low PE content (down from 30% in the ER-like GUVs to 2%), called CIS-GUV, and used it in the TBSV replicase assembly assay. As expected, production of new (+)repRNA was significantly reduced when CIS-GUV with low PE content was used (Fig. 10C, lane 3) (175). However, the production of recRNAs and short aberrant RNAs was reduced below detection level (Fig. 10C). Based on these data, we suggest that high PE content in VROs is beneficial for RNA recombination. Thus, our data suggest that the phospholipid composition, especially the PE content, of the membranes used to assemble the viral replicase is a major factor in RNA recombination.

Another important phospholipid in VROs and autophagy membranes is the minor phospholipid PI(3)P (176), which is enriched in VROs and required for TBSV replication *in vitro*, in yeast and plants (99, 163, 175, 177). The subverted ATG2 and the autophagy pathway are critical for PI(3)P enrichment in VROs (99, 101). In addition, the co-opted ATG11 autophagy protein was shown to be involved in sterol enrichment of VROs (164). To test the role of PI(3)P and sterol in TBSV recombination, we added 1% PI(3)P and 5% ergosterol to the ER-like GUV preparation. This is because the ER membrane is poor in PI(3)P and sterol contents (178), whereas the VRO’s membrane is enriched in PI(3)P and sterol (164, 177, 179, 180). Then, functional TBSV replicase was assembled using the GUV preparation in combination with purified recombinant MBP-p33, MBP-p92, TBSV repRNA, and SF-CFE soluble cytosolic fraction obtained from yeast. The control was prepared with the same procedure except omitting PI(3)P and ergosterol from the GUV preparation with ER-like lipid composition. The four ribonucleotides, including ^32^P-labeled UTP, were added to start the *in vitro* replication assay. Gel analysis showed that the replicase assembled with GUV containing PI(3)P and ergosterol supported low RNA recombination (Fig. 10D). However, time course experiments led to the detection of short aberrant RNA products with ~2fold increased efficiency with PI(3)P-sterol-containing GUVs when compared with the ER-like GUVs (Fig. 10D). dsRNA replication intermediate and the new (+)RNA progeny, the authentic replication products, were also increased when PI(3)P and ergosterol were present in GUVs, as observed previously (175). Altogether, these *in vitro* data suggest that the enrichment of PI(3)P, a signature lipid of the autophagosomal membrane (181) and the VRO (163, 177), and sterols promote not only authentic replication but also the production of aberrant (truncated) RNA products. These aberrant products might participate in RNA recombination as shown previously with TBSV in yeast (37, 50, 58).

Confocal microscopy images showed that the presence of TBSV p33 and p92^pol^ changed the PE [labeled with 1,2-dioleoyl-sn-glycero-3 phosphoethanolamine-N-(7-nitro-2–1,3-benzoxadiazol-4-yl) (NBD) fluorescent dye] distribution in GUV preparations (Fig. 10F and G) as shown previously (175). Interestingly, the NBD-PE-rich membranes excluded the soluble MBP-BFP, suggesting a restrictive membranous microenvironment induced by TBSV p33 and p92^pol^ replication proteins in GUVs (Fig. 10G, compare left panel with the control right panel).

Because of the co-opted autophagosomal membrane and the hijacked ATG2 autophagy-related lipid transfer protein needed for high PE (and PS and PI(3)P) enrichment in VROs (99, 101), we propose that inhibition of the autophagy pathway leads to reduced TBSV RNA recombination due to the low PE content of VROs in autophagy-deficient or ATG2-depleted host cells. Altogether, the *in vitro* replicase reconstitution data are in line with the ATG2 overexpression results in yeast, supporting the notion that co-opted autophagy affects TBSV RNA recombination via modulating the phospholipid composition of VROs and protein:phospholipid ratio in replicase-containing membranes.

## DISCUSSION

(+)Strand RNA viruses use RNA recombination and high-frequency mutations for rapid evolution and adaptation to new hosts and environments (11–14, 16, 38, 182, 183). Understanding the factors affecting RNA recombination is important, as demonstrated by the recent SARS-CoV-2 pandemic. TBSV and yeast model host serve as a powerful system to dissect several factors affecting virus replication and RNA recombination using yeast genomic and proteomic collections (40, 42, 184, 185). Another intriguing approach is to use bacterial effectors or heterologous viral proteins to alter cellular processes, which could affect TBSV replication and recombination if they target common/shared resources also needed by TBSV (10, 104, 107, 186). We exploited this approach in our work, utilizing the *Legionella* effector library and SARS-CoV-2 and HMPV gene collections. Overall, we identified 16 *Legionella* effectors and 6 SARS-CoV-2 and 2 HMPV proteins, whose expressions in yeast greatly affected TBSV RNA recombination. The identified effectors might affect RNA recombination by targeting co-opted host factors involved in the assembly of the TBSV replicase complexes and viral polymerase function or by altering cellular functions involved in viral restriction. The groups of effectors/viral proteins tested are known to target various conserved cellular proteins and pathways, thus highlighting the possible participation of those cellular factors in viral RNA recombination. Indeed, the role of the actin network in TBSV RNA recombination was characterized before (47), and it has been identified in this work as well, validating our approach. Altogether, this novel approach could lead to rapid identification of host factors/pathways involved in viral RNA recombination in a functional setting.

TBSV RNA recombination is based on not fully random events, but is affected by recombination “hot spots” and cis-acting elements on the RNA templates (29, 38, 53, 111). In addition, host factors and environmental conditions also greatly affect the template-switching events by the viral RdRp (46, 50, 74, 113, 187). Therefore, TBSV RNA recombination could be affected by different host factors from those involved in TBSV replication. Accordingly, our extensive TBSV replication and recombination studies have not revealed a strong correlation between replication efficiency and RNA recombination frequency, except in a few cases. For example, our previous genome-wide screens with knockout, knockdown, and temperature-sensitive yeast libraries covering ~95% of yeast genes on TBSV replication and recombination revealed that most of the identified host factors affecting TBSV replication did not affect TBSV RNA recombination (40, 44, 45, 47, 112, 185). In the case of our *Legionella* effector screens, we found that 28 affected TBSV replication (107), whereas 16 *Legionella* effectors affected TBSV recombination, but only 7 of those affected both TBSV replication and recombination (Table 1). Also, another example is the expression of HMPV P protein (Fig. 3D, lanes 1–2) that does not affect TBSV replication, but enhances recombination by ~3-fold. Expression of Nsp1 of SARS-CoV-2 does not affect TBSV replication (Fig. 3A, lanes 7–8), but enhances recombination by ~6-fold. Rapamycin treatment reduced TBSV replication, but increased RNA recombination (Fig. 9A). These examples showed no strong positive correlation between recombination frequency and replication rate. Although Xrn1 ribonuclease suppresses both TBSV replication and RNA recombination, the mechanisms and targets of Xrn1 are different. During replication, the genomic RNA is targeted by Xrn1, whereas Xrn1 destroys the partially degraded TBSV RNAs (called degRNAs, Fig. 1B) generated by host endonucleases, which are involved in RNA recombination (50). Altogether, based on our previous and current work, we propose that there are unique host factors modulating mostly TBSV RNA recombination, other unique host factors affecting only TBSV replication, whereas a group of host factors could affect both processes. We envision a similar complex picture on the effects of bacterial effectors and heterologous viral proteins on TBSV replication versus RNA recombination.

### Utilizing TBSV-yeast model host as a cellular system sensor to identify novel targets of heterologous pathogenic proteins

**Fig 11:**
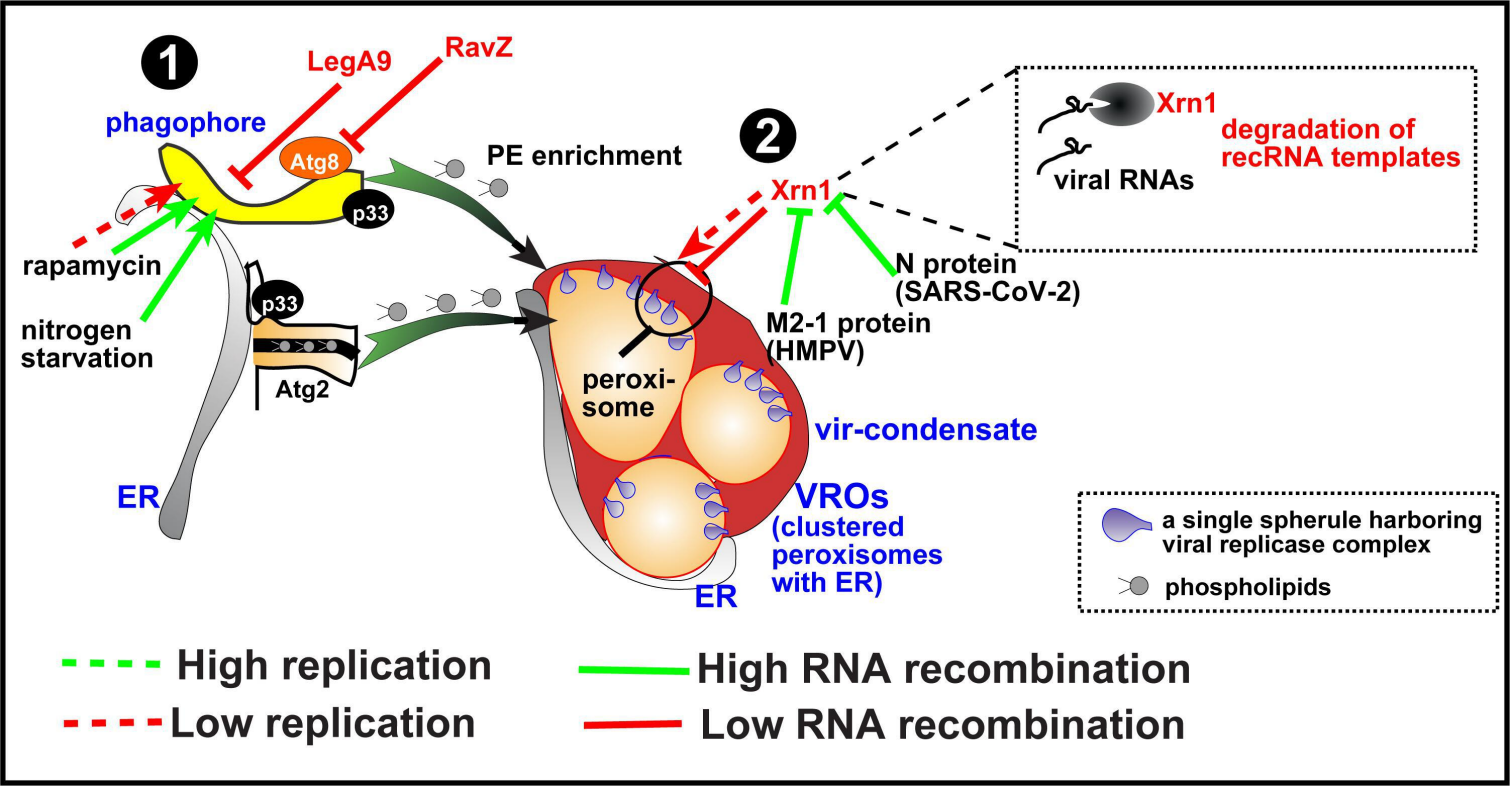
A summary model on the roles of the tested factors on TBSV replication and RNA recombination. #1 scheme shows the pro-recombinational role of co-opted autophagy and ATG2 bulk lipid transfer protein via phospholipid enrichment in VRO (center). The inhibitory *Legionella* RavZ and LegA9 effectors of TBSV RNA recombination are shown in red, and the stimulatory treatments are shown in green. The #2 scheme shows the inhibitory effect of the host Xrn1 5´−3´ exoribonuclease on TBSV replication and RNA recombination. Xrn1 nuclease antiviral activities are prevented by the heterologous SARS-CoV-2 N nucleocapsid protein and the HMPV M2-1 protein, which leads to enhanced TBSV replication and increased recombinant RNA production. The model also illustrates that the TBSV-yeast system could be used as a cellular system sensor to define the targets of heterologous bacterial and viral proteins in a functional assay.

Our novel approach based on the TBSV-yeast system might also be useful to identify new functional targets of pathogenic proteins from bacteria or viruses that are expressed in eukaryotic cells. This was demonstrated by using yeast mutants with known functional deficiencies in TBSV replication or RNA recombination. Specifically, SARS-CoV-2 N protein was shown to enhance TBSV replication and RNA recombination in yeast. Then, we tested the effect of the N protein on TBSV replication and RNA recombination in yeast mutants that were known to affect TBSV (40, 49, 52, 99). Accordingly, we found that yeast lacking the single conserved Xrn1 5´−3´ ribonuclease did not recapitulate the enhancing effect of the N protein on TBSV replication and RNA recombination. Moreover, the expression of the N protein also increased CIRV replication and subgenomic RNA production, which is inhibited by Xrn1 in yeast. Expression of the N protein also enhanced the replication of yeast L-A dsRNA virus, which is sensitive to the antiviral function of Xrn1 (156). The simplest explanation is that the N protein targets/prevents Xrn1 antiviral function in yeast, thus facilitating TBSV and CIRV replication and RNA recombination (Fig. 11). This is supported by our previous findings that the host Xrn1 is a major inhibitor of TBSV replication and RNA recombination in yeast and plants (38, 49, 51, 52, 113).

Interestingly, the N protein partitions into p33-induced vir-condensates associated with TBSV VROs. SARS-CoV-2 N protein is known to form biomolecular condensates in human cells (188), suggesting that it could be prone to partition in cellular condensates. However, the N protein does not affect the TBSV replicase activity *in vitro*. Overall, the inhibitory effect on Xrn1 antiviral function is a new finding for SARS-CoV-2 N protein’s activities. Interestingly, expression of HMPV M2-1 protein also prevented Xrn1 antiviral function in yeast, and M2-1 expression led to enhanced TBSV and CIRV replication and a similar recombination profile to that observed with expression of SARS-CoV-2 N protein in yeast. However, the expression of HMPV P protein showed a unique recombination profile, suggesting a different host target for the P protein, which will need further studies. Altogether, our findings indicate that Xrn1 might be a critical RNA virus restriction factor, and it is likely targeted by SARS-CoV-2 N protein and HMPV M2-1 during viral infections.

Xrn1 has been shown to affect several human and plant viruses. Xrn1 also plays a poorly defined role in SARS-CoV-2 replication based on CRISPR screen (189). Xrn1-produced small RNAs from dengue virus and other flaviviruses (190–192) and plant RNA viruses (193) have pro-viral functions via inhibition of host immune responses. Measles virus subverts Xrn1 to inhibit host immune responses (194). Xrn1 also plays a role in several human diseases (195).

We envision that similar approaches could be used in future studies to identify new functional intracellular targets of various bacterial and heterologous viral proteins based on using the TBSV-yeast model as a sensitive system sensor.

### Role of the autophagy pathway in TBSV RNA recombination

The most frequent target of the expressed *Legionella* effectors and SARS-CoV-2 proteins was the autophagy pathway. Although the pro-viral roles of autophagy proteins and pathway have been recently characterized in TBSV replication in yeast and plants (99–102), its role in viral RNA recombination was not known. Therefore, in this work, we dissected the role of autophagy in TBSV recombination. Inhibition of autophagy by expression of the *Legionella* effectors RavZ and LegA9 inhibited the production of TBSV recombinants in both yeast and plants. Moreover, these effectors inhibited the robust recruitment of ATG8 core autophagy protein into VROs, suggesting the inhibition of subversion of the autophagy proteins/membranes for TBSV VRO biogenesis. On the contrary, induction of the autophagy pathway by inhibition of mTOR kinase by rapamycin or by nitrogen starvation of yeast cells led to enhanced viral RNA recombination. Altogether, these data support the surprising notion that the autophagy pathway plays a pro-recombination role during TBSV replication. Moreover, the first identification of the autophagy pathway’s role in TBSV recombination also demonstrates that bacterial effectors and heterologous viral pathogenicity proteins could be used to discover new aspects of TBSV biology (Fig. 11).

But how could autophagy affect viral RNA recombination? The key data obtained were based on overexpression of ATG2 autophagy protein with known bulk phospholipid transfer function in yeast. The co-opted ATG2 has recently been shown to be involved in the enrichment of PE, PS, and PI(3)P phospholipids in TBSV VROs (101). These phospholipids are critical for the proper function of the viral replicase or the assembly of the replicase complex during TBSV replication (172, 175). Overexpression of ATG2 promotes PE and other phospholipid enrichment in TBSV VROs (101) and stimulates RNA recombination. A GUV-based TBSV replicase assembly system was used to test the effect of the phospholipid composition of the GUV membrane on viral RNA recombination. This novel approach led to the identification of PE, which is enriched and a signature lipid in TBSV VROs, as a pro-recombination factor. High PE content in VROs might promote RNA recombination, because low PE content in GUVs greatly inhibited RNA recombination and short aberrant RNA production *in vitro*. PE is critical for TBSV replication (172, 175, 177) and needed for VRO formation, including the characteristic small membrane invaginations, called spherules, which harbor the viral replicase (162). We found that increasing the ratio of phospholipids versus viral proteins in the GUV-based replicase assembly assay stimulated RNA recombination *in vitro*. This suggests that phospholipids likely have a direct effect on viral replicase template-switching activity. We also found that PI(3)P and sterols in GUVs increased the production of canonical dsRNA and (+)ssRNA progeny, but also short aberrant (truncated) RNAs, which might serve as RNA recombination intermediates (24, 46, 50, 113). PI(3)P might affect RNA recombination by helping recruitment of various pro-viral factors with known PI(3)P-binding abilities, such as selected autophagy proteins, ESCRT proteins, and the retromer-associated sorting Nexin-BAR proteins (99, 161, 180, 196, 197). Overall, this work highlights for the first time the critical role of membrane phospholipids, membrane lipid context, and membrane protein:phospholipid ratio in the regulation of viral RNA recombination. Because all (+)RNA viruses replicate in membranous intracellular structures, the findings with TBSV could be relevant for other viruses of plants, animals, and humans.

### Limitations of this study

Expression of bacterial effectors and heterologous viral pathogenicity proteins in yeast could potentially lead to artifactual changes in some cases that affect TBSV replication and/or RNA recombination, but it may not reflect the effectors’ normal functions in their natural host cells. However, the effects of those effectors on cellular processes could still unravel new biology for TBSV replication and recombination.

## MATERIALS AND METHODS

### Yeast strains and yeast and plant expression plasmids

The yeast (*Saccharomyces cerevisiae*) strain BY4741 (MATa his3Δ1 leu2Δ0 met15Δ0 ura3Δ0), and single gene-deletion YKO strains Δxrn1, Δski2, Δhur1 (renamed as Δpmr1-1), Δatg2, Δatg8, and Δatg9 were obtained from Open Biosystems.

The following yeast expression plasmids have been previously described: the library of pAG416GAL constructs to express 113 *Legionella* effectors was constructed before (107). The following yeast plasmids were constructed before: pGAD-LEU-Cup1-His92 (198), pGBK-His/Cup-His33/GalDIAU, pGBK-HIS-Gal1-BFP-p33/Gal1-DI72 (132). The following plant expression plasmids have been previously described: pGD-p33-GFP, pGD-p33-BFP, pGD-His-p92, pGD-RFP-SKL (132, 172, 199).

Individual SARS-CoV-2 genes (Table 3) were PCR-amplified from plasmids received from Nevan Krogan (134) using primers listed in Table 4.

**TABLE 3:** Plasmids constructed in this study^*a*^

Gene	Primers	Template	Restriction sites (PCR)	Restriction	Restriction site (plasmid)
Nsp4	C1/C2	pLVX-EF1alpha-nCoV2019-nsp4-2xStrep-IRES-Puro	BglII-SalI	pYES/NT/C	BamHI-XhoI
Nsp1	C1/C5	pLVX-EF1alpha-nCoV2019-nsp1-2xStrep-IRES-Puro	EcoRI-BamHI	pJP201	EcoRI-BamHI
Nsp3	C12/C13	pDONR207 SARS-CoV-2 NSP3 (Addgene, cat# 141257)	BglII-XhoI	pYES/NT/C	BamHI-XhoI
Nsp5	C1/C8	pLVX-EF1alpha-nCoV2019-nsp5-2xStrep-IRES-Puro	EcoRI-BamHI	pJP201	EcoRI-BamHI
Nsp15	C1/C20	pLVX-EF1alpha-nCoV2019-nsp15-2xStrep-IRES-Puro	EcoRI-BamHI	pJP201	EcoRI-BamHI
Orf7b	C1/C33	pLXV-EF1a-IRES-Puro_2xStrep-nCoV2019-orf7b	EcoRI-BamHI	pJP201	EcoRI-BamHI
N	C1/C23	pLVX-EF1alpha-nCoV2019-N-2xStrep-IRES-Puro	EcoRI-BamHI	pJP201	EcoRI-BamHI
N	C39/C46	pLVX-EF1alpha-nCoV2019-N-2xStrep-IRES-Puro	BamHI-XhoI	pJP202	BamHI-XhoI
DNa/DC	C40/C45	pLVX-EF1alpha-nCoV2019-N-2xStrep-IRES-Puro	BamHI-XhoI	pJP202	BamHI-XhoI
Dlinker	C39 /(C47) + (C48)/C46	pLVX-EF1alpha-nCoV2019-N-2xStrep-IRES-Puro	BamHI-XhoI	pJP202	BamHI-XhoI
NTD + CTD	C40 /(C47) + (C48)/C45	pLVX-EF1alpha-nCoV2019-N-2xStrep-IRES-Puro	BamHI-XhoI	pJP202	BamHI-XhoI
NTD + linker	C40/C43	pLVX-EF1alpha-nCoV2019-N-2xStrep-IRES-Puro	BamHI-XhoI	pJP202	BamHI-XhoI
Nterm + linker	C39/C43	pLVX-EF1alpha-nCoV2019-N-2xStrep-IRES-Puro	BamHI-XhoI	pJP202	BamHI-XhoI
NTD	C40/C41	pLVX-EF1alpha-nCoV2019-N-2xStrep-IRES-Puro	BamHI-XhoI	pJP202	BamHI-XhoI
CTD	C44/C45	pLVX-EF1alpha-nCoV2019-N-2xStrep-IRES-Puro	BamHI-XhoI	pJP202	BamHI-XhoI
HMPV	M2-1 7925/7926	pHMPV M2-1	BamHI-XhoI	pJP202	BamHI-XhoI
HMPV	M2-1/C21S 8316/7926 + 8317/7926	pHMPV M2-1	BamHI-XhoI	pJP202	BamHI-XhoI
HMPV *P*	7905/7906	pHMPV *P*	BamHI-XhoI	pJP202	BamHI-XhoI
HMPV F TN-96	8007/8004	pHMPV F TN-96	BglII-XhoI	pEsc/Cup	BamHI-XhoI
Nsp1	C1/C2	pLVX-EF1alpha-nCoV2019-nsp1-2xStrep-IRES-Puro	BglII-SalI	pMalc-2x	BamHI-SalI
RFP-N	C1/C23	pLVX-EF1alpha-nCoV2019-nsp1-2xStrep-IRES-Puro	BamHI-XhoI	pKai240	BamHI-SalI
RFP-M2-1	7925/7926	pHMPV M2-1	BamHI-XhoI	pKai240	BamHI-SalI
N	C1/C23	pLVX-EF1alpha-nCoV2019-N-2xStrep-IRES-Puro	EcoRI-BamHI	pJP203	EcoRI-BamHI

^*a*^ + implies sequential PCR steps.

**TABLE 4:** Primers used in this study

Primer designation	Primer sequence
C1	CCGAGATCTCAAGAATTCGCCGCCACCATG
C2	CCAGTCGACGGAGAGGGGCGGGATCC
C5	CCGGGATCCTTAGCCGCCGTTCAGTTCGCGCATC
C8	CCGGGATCCTTACTGGAAAGTGACCCCACTG
C12	CCGAGATCTATGGCCCCTACCAAGGTGACC
C13	CCGCTCGAGTTATCCGCCCTTCAGGGCG
C20	CCGGGATCCTTATTGCAACTTTGGATAGAAGGTCTCG
C23	CCGGGATCCTTACGCCTGAGTAGAATCGGC
C33	CCGGGATCCTTAGGCGTGGCATGTCTCGTTATGGTC
C39	CCAGGATCCATGAGCGATAACGGCCC
C40	CCAGGATCCATGGGATTGCCAAATAACACAGC
C41	CCGCTCGAGCTAGCTGCCGCCGCGTGATCCTTC
C42	CCAGGATCCATGCAAGCATCCTCAAGGTCTAG
C43	CCGCTCGAGCTATACGGTTTGACCTTGTTGCTG
C44	CCAGGATCCATGACCAAGAAAAGCGCTGCAGAAG
C45	CCGCTCGAGCTATGGAAACGTCTTATACGCGTC
C46	CCGCTCGAGCTACGCCTGAGTAGAATCGGCTG
C47	CCAGCTAGCGCTGCCGCCGCGTGATCCTTC
C48	CCAGCTAGCACCAAGAAAAGCGCTGCAGAAG
7905	GGCAGAATTCATGTCATTCCCTGAAGGAAAAG
7906	GGCACTCGAGCTACATAATTAACTGGTAAATG
7925	CCAGGGATCCATGTCTCGTAAGGCTCCATGC
7926	CCAGCTCGAGTTACTGCACTTGATTAGTGC
8004	GGCACTCGAGCTAATTATGTGGTATGAAGCCATTG
8007	CGAGATCTATGTACCCATACGATGTTCCAGATTACGCTGGATCCATGTCTTGGAAAGTGG
8316	CGGGATCCATGTCTCGTAAGGCTCCATGCAAATATGAAGTGCGGGGCAAATGCAACAGAG
8317	GGGCAAATGCAACAGAGGGAGTGATTCCAAATTCAATCACAATTACTGGAGTTGGCCTG
8170	GTAATACGACTCACTATAGGAGAAGCACGCAACATGTTCATC
5440	CCAGCTCGAGGAAAAATTTTTAAATTCATATAACTCCC

The SARS-CoV-2 *NSP3* gene was PCR-amplified from pDONR207 SARS-CoV-2 NSP3 (cat# 141257, Addgene). Individual HMPV genes were PCR-amplified using primers listed in Table 4 from plasmids kindly provided by Dr. Becky Dutch. The polylinker of pYES/NT/C was modified to obtain pJP201 by first PCR-amplifying the *NSP4* gene from plasmid pLVX-EF1alpha-nCoV2019-nsp4-2xStrep-IRES-Puro with forward primer C1 (containing BglII and EcoRI sites) and reverse primer C2 (containing BamHI and SalI sites). The PCR product was digested with BglII and SalI and cloned into the BamHI and XhoI sites of pYES/NT/C. The reengineered EcoRI and BamHI sites were used for further cloning.

In order to introduce an HA-tag at the N terminus of proteins, first, HMPV fusion protein TN96 was PCR-amplified with forward primer #8007 and reverse primer #8004, followed by digestion with BglII and XhoI, and the PCR product was cloned into BamHI/XhoI-digested pEsc-Ura/Cup to produce pJP202. The reengineered BamHI and XhoI sites were used for further cloning.

His-tagged N protein mutants (Fig. 4A) were constructed using PCR amplification with primers (listed in Table 3) using pYES/NT/C yeast plasmid digested with BamHI and XhoI.

For the expression of N and M2-1 proteins as tagRFP-fusion proteins in plants (RFP is at the N terminus), we constructed pGD/tagRFP/N and pGD/tagRFP/m2-1 by PCR amplification of SARS-CoV-2 N and HMPV M2-1 genes as described above, followed by digestion with BamHI and XhoI and cloning into pKai240 digested with BamHI and SalI.

In order to express the SARS-CoV-2 genes in *E. coli* with an N-terminal MBP-tag, first, the *NSP1* gene was PCR-amplified from pLVX-EF1alpha-nCoV2019-nsp1-2xStrep-IRES-Puro using forward primer C1 and reverse primer C2. The PCR product was digested with BglII and SalI and cloned into the BamHI and Sal1 sites of pMALc-2X to produce pJP203. The reengineered EcoRI and BamHI sites were used for further cloning.

In order to clone the full-length genomic RNA of CIRV into a yeast expression plasmid, first, the CUP1 promoter upstream of the transcription site was PCR-amplified and ligated to a PCR product corresponding to the full-length cDNA of gCIRV. The ligated PCR products were PCR-reamplified with the end primers and digested with BamHI-XhoI to replace the promoter and the insert of pGADCupHisCNV92 (45). Since the XhoI sequence is present in the CIRV DNA sequence, the previous digestion removed the 3´ end of the genome. This was corrected by reintroducing this portion, together with a 3´ ribozyme (39), to obtain a copper-inducible full-length cDNA of the CIRV genomic RNA with a ribozyme behind the 3´ end of the CIRV sequence.

### Screening of *Legionella* effectors and heterologous viral proteins in a TBSV recombination assay in yeast

To measure the effects of *Legionella* effectors on TBSV replication and RNA recombination, the yeast strain BY4741 was transformed with pGBK-His/Cup-His33/GalDIAU and pGAD-LEU-Cup1-His92 and each of the 113 pAG416GAL-effector constructs to express *Legionella* effectors separately (107). The transformed yeast strains were grown in synthetic complete medium without uracil, leucine, and histidine (SC-ULH^-^) medium supplemented with 2% glucose and 100 µM bathocuproinedisulfonic acid (BCS) in 96-deepwell plates at 29°C for 16 h. To induce *Legionella* effector expressions, the yeast strains were cultured in SC-ULH^-^ medium supplemented with 2% galactose and 100 µM BCS at 23°C for 8 h. And then, the yeast strains were grown in SC-ULH^-^ medium supplemented with 2% galactose and 50 µM CuSO_4_ at 23°C for 36 h. After the steps, yeast strains were harvested for RNA or protein analysis (198).

The pGAD-LEU-Cup1-His92 and pGBK-His/Cup-His33/GalDIAU and either pAG416GAL-RavZ or pAG416GAL-LegA9 or pAG416GAL (control) plasmids were transformed into BY4741. For the expression of RavZ and LegA9, the transformed yeast strains were grown as described above. After the harvest, RNAs and proteins were analyzed (166).

The effects of SARS-CoV-2 and HMPV proteins on TBSV recombination were tested by introducing pGAD-Leu/Cup-His92 and pGBK-His/Cup-His33/GalDIAU and pEsc-Ura/Cup/HA/GOI (HA-tagged gene-of-interest), pYes-Ura/Gal/His/GOI (His_6_-tagged gene-of-interest), or pEsc-Ura/Cup/GOI (non-tagged version) via transformation into BY4741 yeast. The yeast assays were done as described above, except the culturing was at 29°C for 24 h.

Nitrogen starvation (200) was induced by inoculating freshly transformed and streaked yeast from ULH^-^ glucose agar media directly into nitrogen-starvation media with copper (0.17% yeast nitrogen base without amino acids and ammonium sulfate and 2% glucose or 2% galactose). Autophagy was also induced by adding 100 ng/mL rapamycin (166) into the ULH^-^gal/copper media after pregrowth overnight in ULH^-^ glucose with BCS (see above).

For FHV replication, the tombusvirus plasmids were substituted with pEsc-His/Cup/FHV/RNA1 (41) and pEsc-Leu/Cup/empty, and the effect of SARS-CoV-2 N protein on FHV replication was tested as above.

For yeast L-A virus replication, BY4741 yeast carrying endogenous L-A virus and expressing SARS-CoV-2 N protein or empty vector (control) were grown in ULH^-^ galactose media containing Cu_2_SO_4_ for 40 h at 29°C, followed by isolating total RNA preparations (24). Digoxigenin (DIG)-labeled RNA probe (75) for the detection of L-A virus was generated from a T7 promoter-containing PCR product, which was obtained by reverse transcription with primer #8170 and PCR with #5440 and #8170.

### *In vitro* TBSV replication assay

MBP-p92^pol^ and MBP-p33 and MBP-N protein and MBP were expressed in *E. coli*, and protein purification was carried out as described (201). Cell-free extracts (CFEs) were prepared from the BY4741 yeast strain as described earlier (187). The CFE-based reaction mixtures were programmed with 0.5 µg DI-72 (+)RNA transcripts and 200 ng affinity-purified MBP-p92^pol^ and MBP-p33 as described (187). The recombinant proteins, MBP-N protein or MBP (as a control) (400 ng, each), were added to the reaction mixtures. The reactions were performed for 3 h at 25°C. The obtained ^32^P-labeled RNA products were separated with electrophoresis in semi-denaturing polyacrylamide gel containing 8 M urea with 0.5× Tris-borate-EDTA buffer (187).

### Analysis of TBSV recombination in plants

Transient expression in *N. benthamiana* leaves was performed by agroinfiltration (202). pEarlygate-RavZ or pEarlygate-LegA9 constructs were transformed into *Agrobacterium tumefaciens* C58C1 (107, 203). One day after the agroinfiltration of pEarlygate-RavZ or pEarlygate-LegA9 plasmids in combination with pGD-p19 plasmid (a suppressor of gene silencing), the infiltrated leaves were agroinfiltrated with pGD-p92, pGD-p33, and pGD-DIAU and harvested 2-day afterward for RNA and protein analysis (107).

### Confocal microscopy analysis of plants

To observe the effect of RavZ or LegA9 on co-localization of TBSV p33 replication protein and ATG8 or ATG11 in *N. benthamiana,* leaves were co-agroinfiltrated with pGD-p33-BFP, pGD-RFP-SKL, pGD-GFP-ATG8, or pGD-GFP-ATG11 (99, 164) and pEarlygate-RavZ or pEarlygate-LegA9 in combination with pGD-p19 into *N. benthamiana* leaves. The leaves were sap-inoculated with TBSV 1 day after agroinfiltration. The confocal microscopy images were taken 2.5 days after agroinfiltration. The confocal microscopy images were obtained sequentially with an Olympus FV1000 microscope (Olympus, America) and merged using FV10-ASW4.2 (107).

### FRAP

Agroinfiltration of *N. benthamiana* with pGD-p33-BFP, pGD-N-RFP, or pGD-M2-1 and pGD-p19 plasmids was done according to reference 75, which was followed 2.5 days later by a treatment with 10 µM latrunculin B (Abcam) to avoid the movement of VROs (at least 3 h before microscopic imaging) (75). FRAP assay was performed with Olympus FV3000 microscope as described (75). Photobleaching was performed in the middle area of VROs. The photobleaching process lasted ~4–5 seconds with a 405 nm laser at 80% intensity. The calculation method for recovery rate was conducted according to reference 75.

### Preparation of GUVs

A total of 1 mg/mL solutions of phospholipid mixtures in chloroform, which were prepared from 25 or 10 mg/mL stock solutions (Avanti, USA) in chloroform and stored at −20°C under nitrogen gas, was used for GUV preparation. Vesicle Prep Pro (Nanion, Germany) workstation was used for the preparation of GUVs by electroformation as described (175). At the end of the protocol, sorbitol solutions containing GUVs were pipetted into an Eppendorf tube and stored at 4°C for 1–2 weeks. We prepared GUVs with “minimal” phospholipid composition (45% PC, 30% PE, 25% PS), with “ER-like” lipid composition (46% PC, 30% PE, 13% PI, 5% PG, 3% PS, 3% CA) or with “*CIS*” phospholipid composition (66% PC, 22% PI, 12% PS + 2% NBD-PE) as described (175).

### Reconstitution of the TBSV replicase in the GUV-based assay

A procedure described earlier (175) was used to perform the replicase assembly assay using GUVs. Briefly, the SF-CFE fraction of yeast was added to a reaction mixture containing all the components: 0.25 µg DI-72 (+)repRNA transcripts, 200 ng *E. coli*-expressed and affinity-purified recombinant MBP-p33, 200 ng recombinant MBP-p92^pol^, 30 mM HEPES-KOH, pH 7.4, 150 mM potassium acetate, 5 mM magnesium acetate, 0.13 M sorbitol, 0.4 µL actinomycin D (5 mg/mL), 2 µL of 150 mM creatine phosphate, 0.2 µL of 10 mg/mL creatine kinase, 0.2 µL of RNase inhibitor, 0.2 µL of 1 M dithiothreitol, 2 µL of rNTP mixture (10 mM ATP, CTP, and GTP and 0.25 mM UTP/^32^P-UTP), and aliquoted GUVs. The assays were performed at 25°C for 2.5 h. The ^32^P-labeled repRNA products synthesized in the replication assay were loaded without heat treatment onto the 5% polyacrylamide gel containing 8 M urea and separated by electrophoresis in 0.5× Tris-borate-EDTA buffer (197).

### GUV analysis by confocal laser microscopy

To visualize GUVs in the presence of viral components, we used a fluorescent PE [18:1 NBD-PE (1,2-dioleoyl-sn-glycero-3 phosphoethanolamine-N-(7-nitro-2–1,3-benzoxadiazol-4-yl))] (Avanti Polar lipids, Inc.), which was dissolved in dimethyl sulfoxide (DMSO) in 8 mM concentration (204) and stored at −20°C. GUVs containing NBD-PE were analyzed after replicase assembly assay (2 h incubation, see above) using 488 nm laser (GFP channel) in an Olympus FV1000 confocal laser scanning microscope.

## Data Availability

The data supporting the conclusions of this article are included within the article or are available from the authors upon request.
